# m6A-lncRNA landscape highlights reduced levels of m6A modification in glioblastoma as compared to low-grade glioma

**DOI:** 10.1186/s10020-025-01254-x

**Published:** 2025-05-17

**Authors:** Giedrius Steponaitis, Rugile Dragunaite, Rytis Stakaitis, Amit Sharma, Arimantas Tamasauskas, Daina Skiriute

**Affiliations:** 1 Laboratory of Molecular Neurooncology, Neuroscience Institute, Lithuanian University of Health Sciences, Kaunas, Lithuania; 2 Department of Stereotactic and Functional Neurosurgery, University Hospital of Bonn, Bonn, Germany; 3 Department of Integrated Oncology, Center for Integrated Oncology (CIO), University Hospital Bonn, Bonn, Germany

**Keywords:** N6-methyladenosine (m6 A), LncRNAs, DRNA-seq, Glioma, Survival

## Abstract

**Background:**

Efforts to understand the interplay between m6A (N6-methyladenosine) modification and long non-coding RNAs (lncRNAs) in the pathogenesis of various diseases, including cancer, have recently attracted considerable attention.

**Methods:**

Herein, we profiled epitranscriptome-wide m6A modifications within lncRNAs at single m6A site resolution across different grades of gliomas (Glioblastomas (GB): *n* = 17, Low grade gliomas (LGG): *n* = 9) using direct RNA long-read sequencing.

**Results:**

Our analysis demonstrated that, 1) 98.5% of m6A-modified RRACH motifs were present within mRNA transcripts, while only 1.16% were conspicuous within lncRNAs. Importantly, LGGs exhibited a higher m6A abundance (23.73%) compared to the GB transcriptome (15.84%). 2) The m6A profiles of lncRNAs differed significantly between gliomas, with unsupervised cluster analysis revealing two clusters (C1, C2). LGG dispersed between C1 and C2 clusters while GB stayed mainly in C1. Clinical feature association analysis between m6A clusters showed the tendency of m6A to be associated with higher malignancy grade (*p* = 0.053), while significant association was observed with higher Ki-67 proliferation index (*p* = 0.04), and tumor location (*p* < 0.01). Specifically, brain tumors located in cerebellum (*n* = 3) were highly m6A modified on lncRNAs as compared to tumors in other locations (frontal lobe, *n* = 5, *p* = 0.003; frontotemporal lobe, *n* = 2, *p* = 0.08; occipital, *n* = 2, *p* = 0.038; parietal, *n* = 2, *p* = 0.007; temporal, *n* = 11, *p* < 0.001). Cox regression analysis showed that the status of lncRNAs m6A modifications had no significant value in predicting post-surgical survival time in our GB or LGG cohorts. The trend of higher lncRNA expression in m6A methylated group was observed for the majority of lncRNAs, while only MIR9-1HG (*r* = 0.439, *p* = 0.028) and ZFAS1 (*r* = 0.609, *p* < 0.05) m6A showed statistically significant positive correlations in gliomas. A high-resolution m6A study revealed that mRNA levels of m6A writers and erasers in gliomas do not reflect global m6A methylation.

**Conclusions:**

Overall, we provide evidence that m6A lncRNAs are strongly modulated in gliomas, representing biologically distinct subgroups. Ten novel differentially methylated lncRNAs were identified in gliomas, which might exert regulatory role in glioma cells. These findings may provide a basis for further deeper research on the role of m6A lncRNAs in gliomas.

**Supplementary Information:**

The online version contains supplementary material available at 10.1186/s10020-025-01254-x.

## Background

The contribution of multiple mechanisms in the interplay of epigenome and epitranscriptome in regulating gene expression is evident from the multifaceted role of N6-methyladenosine (m6 A) modifications that modulate not only mRNAs but also non-coding transcripts. Particularly for long non-coding RNAs (lncRNAs), where this combinatorial effect of m6 A and lncRNAs paves a new direction for the study of the underlying regulatory mechanisms of gene expression in disease progression. Being one of the most abundant and well-studied epitranscriptomic marks in higher eukaryotes, sufficient evidence exists for the role of m6 A in RNA metabolism, including RNA structure formation, splicing, polyadenylation, transport, localization, and stability (Cappannini et al. 2023; Kadumuri and Janga 2018; Frye et al. 2018). Primarily, 20 to 40% of all transcripts encoded by mammalian cells contain m6 A methylation, and methylated RNAs usually include multiple m6 A sites per transcript (Dominissini et al. 2012). Comparing the m^6^A methylome between mRNA and lncRNA, mRNA has a higher intensity and average m^6^A peak numbers than lncRNA (Liu et al. 2020a). m6 A RNA modifications are introduced co- or post-transcriptionally (McCown et al. 2020; Boccaletto et al. 2021) on transcripts by modification enzymes, known as “writers”. In eukaryotes, m6 A is positioned at the mRNA nucleotide consensus sequence 5’-RRACH-3’ (R = purine, H = A, C, or U nucleotides) by protein complexes consisting of METTL3/14 (methyltransferase-like protein 3 and 14) and adaptor proteins such as WTAP (Wilms Tumor 1-Associating Protein) (Liu et al. 2014; Ping et al. 2014), VIRMA (Liu et al. 2018), and RBM15 (RNA binding motif protein 15/15B) (Dominissini et al. 2012; Shi et al. 2019; Zaccara et al. 2019). Here it is worth mentioning the relative contribution of other components that leads to m6 A modifications to occur, specially m6 A methyltransferases called “writers”; m6a demethylases called “erasers” and m6 A-binding proteins called “readers”. For instance, demethylases such as FTO (fat-mass and obesity associated protein) and ALKBH5 (alkB homolog 5) act as m6 A mark removers or “erasers” (McCown et al. 2020; Shi et al. 2019; Wei and He 2021). Equally, m6 A can directly and indirectly affect the binding of “reader” proteins (proteins containing the YTH domain), such as YTH domain family 2 (YTHDF2), to human methylated mRNAs and ncRNAs in a cell type-specific manner (McCown et al. 2020; Patil et al. 2018; Wang et al. 2014). It has been discussed that the densely populated m6 A regions could be more frequently occupied by m6 A readers (Shi et al. 2019). Evidence suggests that global m6 A and the expression levels of m6 A regulators, including writers, readers, and erasers, are frequently dysregulated in various types of cancers and are critical for cancer development, progression and drug resistance (Huang et al. 2020).

As aforementioned, m6a can regulate non-coding transcripts (in addition to mRNA), and the interplay between m6a and lncRNAs in this context is notable. For example, in metastasis-associated lung adenocarcinoma transcript 1 (MALAT1), which is frequently upregulated in cancer, m6 A destabilizes the hairpin stem structure of the transcript (Baspinar et al. 2020; Liu et al. 2013) and likely controls the function of MALAT1 in splicing and transcription by regulating RNA–protein interactions (Fazi and Fatica 2019; Zhou et al. 2016). Likewise, the LncRNA XIST (X-inactive specific transcript) is another highly m6 A methylated molecule with at least 78 N6-methyladenosine (m6 A) residues in human cells (Patil et al. 2016). Interestingly, the authors reported that m^6^A methylome in mRNAs and LncRNAs is tissue specific in brain tissues demonstrating the strongest tissue specificity (Liu et al. 2020a). Gliomas are brain tumors that arise from glial cells and include IDH wild-type (IDHwt) glioblastomas (GB), which are considered one of the most common and aggressive tumors in adults, with a median survival of only 8 to 9 months after diagnosis (Ostrom et al. 2023). The course of GB is largely unknown and results in a lack of prevention methods, late diagnosis, and unsuccessful treatment (Alexander and Cloughesy 2017). Recently, several studies have indicated that RNA m6 A methylation is important for tumorigenesis, self-renewal, and therapeutic resistance in GB (Zhang et al. 2017; Cui et al. 2017), and even m6 A-autophagy-lncRNA based prognostic signatures have been proposed (Sharma et al. 2023). Nevertheless, most studies are focused on mRNAs, and there is a lack of studies on m6 A modifications in long noncoding RNAs in clinical brain tumor samples.

In our comprehensive analysis, we identified the fraction of m6 A modified lncRNAs across glioma transcriptome that stratify patient gliomas into two subpopulations distinguishing vast majority of glioblastomas (88%) from subpopulation of low-grade gliomas. Contrary to GB specific feature of heterogeneity, m6 A clustering fused GBs at single pool while LGGs were divided across two clusters. The latter phenomenon forms the basis for the statement of m6 A as GB pathology connecting factor which needs to be confirmed in future research.

Furthermore, we showed that mRNA expression levels of m6 A writers and erasers in gliomas do not reflect global RNA methylation (m6 A), thus future studies based mainly on these components need to be more directive.

## Materials and methods

### Patient samples

A total of 17 glioblastoma (GB) (11 females and 6 males with the median age 67.4 yr (from 50.1 yr to 85.4 yr) and 9 diffuse astrocytoma (LGG) (7 females and 2 males with the median age 33.47 yr (from 24.2 yr to 71.6 yr) tumor tissues were collected at the Department of Neurosurgery, Lithuanian University of Health Sciences Hospital (Kaunas, Lithuania), from 2002 to 2021. The basic clinical characteristics of gliomas are presented in Suppl. Table St1.

### RNA extraction from tumor tissue

Total RNA was isolated from approximately 100 mg of snap-frozen (frozen immediately after surgery and stored in liquid nitrogen) tumor tissue with TRIzol following the manufacturer’s instructions (Invitrogen, cat. no. 15596026). The quality of the isolated RNA was assessed on a 1.5% agarose gel based on the relative abundance of the 18S and 28S rRNA subunits, while the approximate quantity and purity were measured with a NanoDrop 2000 spectrophotometer (Thermo Fisher Scientific).

### PolyA RNA enrichment

On average, 84.6 µg of total RNA was enriched for poly-A tailed RNAs using a Dynabeads™ mRNA DIRECT™ purification kit (Invitrogen™, cat. no. 61012). Next, the poly-A enriched RNA was precipitated overnight at −80˚C in precipitation buffer (final concentrations: 10% 3 M sodium acetate (pH 5.52) (Sigma-Aldrich, cat. no. 32319-500G-R), 100 µg/ml glycogen (Thermo Scientific™, cat. no. R0551), and 2.5 vol of pure ethanol). Finally, the precipitated RNA was resuspended in 15 µl of RNase-free water and evaluated using an RNA 6000 Pico kit (Agilent, cat. No. 5067–1513) on a 2100 Bioanalyzer instrument (Agilent, cat. No. G2939BA).

### RNA library preparation and sequencing

Sequencing libraries were constructed from 600 ng of poly-A-enriched RNA using a direct RNA sequencing kit (Oxford Nanopore Technologies (ONT), cat. no. SQK-RNA002) and following the manufacturer’s protocol (ver. DRS_9080_v2_revO_14 Aug2019). The only correction to this protocol was made by replacing the original reverse transcription adapter (RTA) with a barcoded RTA, designed by Hyeshik Chang^1^. All the samples were divided into groups of 4, except for 2 glioblastoma samples, which were grouped together. Samples in each group were individually barcoded during library preparation and sequenced together. The barcoded samples (50 ng each) were pooled after the reverse transcription reaction (after step 8 in the protocol). Next, the purified libraries were quantified with Qubit™ dsDNA HS assay kit (Invitrogen™, cat. No. Q32851) on a Qubit™ 4 Fluorometer (Invitrogen™, cat. No. Q33238). Finally, 200 ng of libraries were sequenced for approximately 48 h on a R9.4.1 flow cell (ONT, cat. No. FLO-MIN106D) using MinION or MinION Mk1 C sequencers. The initial sequencing quality control consisted of an estimation of 3’-end bias, RNA fragmentation, and base calling error rate. To estimate the extent of 3’-end bias in direct RNA sequencing, gene body coverage was analyzed. On average, the first 10% of the 3’-end sequences of the genes had a 4.11 times higher coverage than the 10% of the 5’-end of genes. To determine the severity of RNA fragmentation, the median read length was compared among samples and between sample groups. The average total read length was 601.7 nucleotides (nt), 605 nt in LGG group, and 599.9 nt in GB group. Finally, read quality scores were compared between samples – the average of all samples was 10.5, 10.1 in LGG group, and 10.7 in GB group, see suppl. fig S20.

### Data analysis

Fast5 files were base-called with high accuracy mode-on using ONT’s Guppy software (ver.: v5.0.11) by applying a configuration file for dRNA-seq (“rna_r9.4.1_70bps_hac.cfg”). The base-called fast5 files were processed with Poreplex software (ver.: 0.5) to extract fastq files for each barcoded sample. Next, the fastq files were aligned to the full Ensembl reference transcriptome (GRCh38.p13; ver.: 105; cDNA + ncRNA) (Cunningham et al. 2022) using Minimap2 software (Settings: *-ax map-ont -p 0 -N 10*; ver.: 2.17-r941) (Li 2018). The aligned SAM files were sorted and indexed with SAMtools (ver.: 1.15.1) (Danecek et al. 2021). The identification of m6 A modifications within RRACH motifs Epinano (ver.:1.2.0) (Liu et al. 2019). Sequencing data were additionally processed using the “nf-core/nanoseq” pipeline (r 3.0.0) (Ewels et al. 2020). Quantitative expression differences between GB and LGG samples, were evaluated with PyDESeq2 software (Muzellec et al. 2023). Agglomerative clustering applying Ward´s linkage for clusters combining Euclidean metrics for similarity analysis between objects was used for hierarchical clustering. Differences between two independent groups were analyzed with a t-test. The chi-square test was used for categorical data analysis. Survival analysis was performed with the Kaplan–Meier method, and the log-rank test was used to compare differences in survival curves across groups. To show the reliability of the survival estimates, confidence intervals (CIs) with 95% confidence level were calculated. The level of significance was *p* < 0.05. Data visualization, target selection, m6 A modification and expression level comparisons were performed using the machine learning and data visualization toolkit “Orange” (ver.3.32, University of Ljubljana). IBM SPSS Statistics (ver.: 27.0.1.0, Armonk, NY, USA) and GraphPad Prism (ver.: 6.01, GraphPad Software, Inc., San Diego, CA, USA) software were used for statistical analysis and data visualization.

## Results

### m6 A epitranscriptome landscape in glioma

Here, we report transcriptome-wide, m6 A posttranscriptional modifications in gliomas. In total, 26 primary human gliomas were selected: 9 grade 2 diffuse astrocytomas (here low-grade gliomas (LGG), median age: 33.37 years (from 24.2 yr. to 71.6 yr.; 77.8% female)) and 17 *IDH* wild-type (IDH*wt*) glioblastomas (GB, grade 4, median age: 67.84 (from 50.1 yr. to 85.4 yr.); 64.7% female)).

**Fig. 1:**
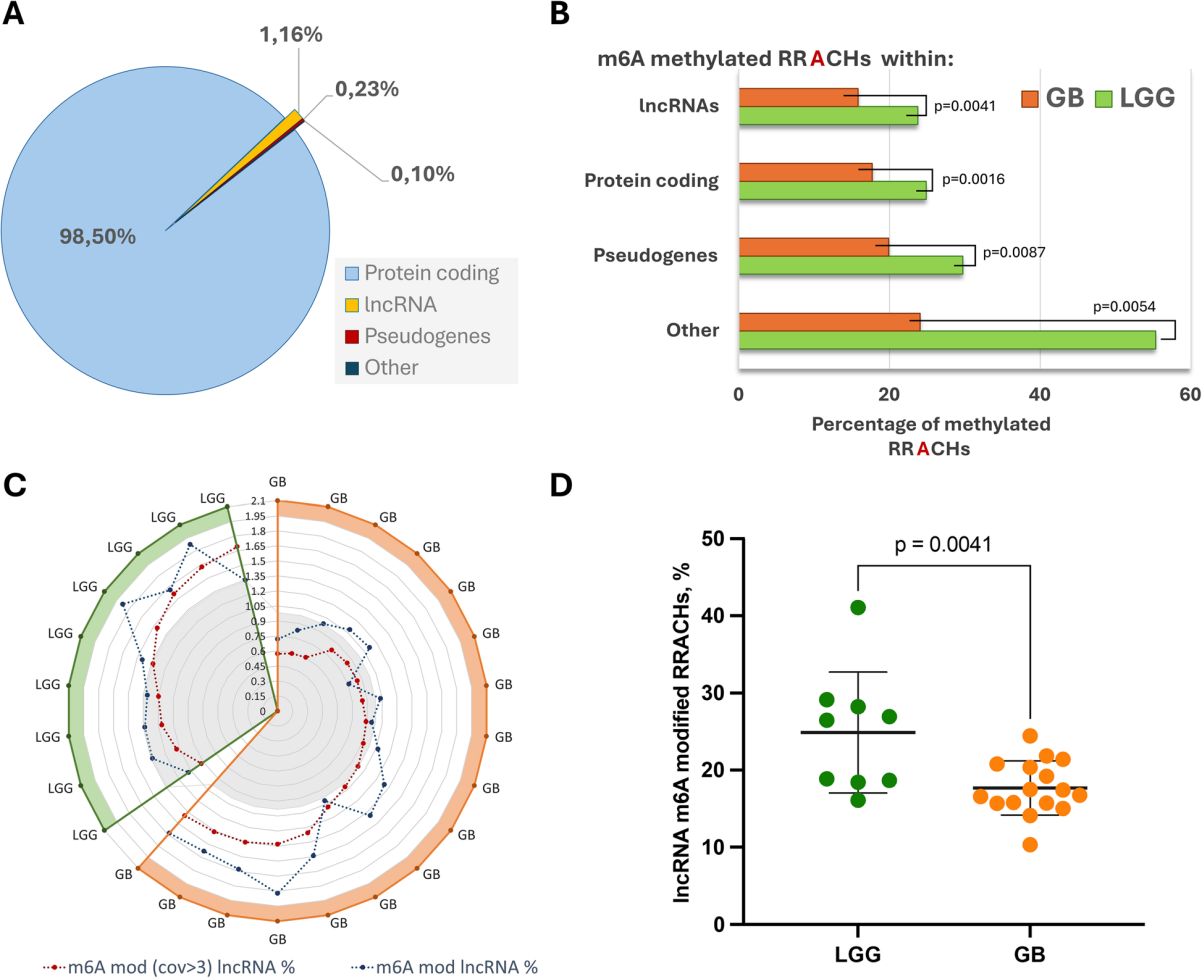
Quantification of m6 A in the glioma transcriptome. **A** The average proportion of identified RRACH motifs in different gene biotypes in gliomas (*n* = 26). **B** A comparison between methylated adenines within RRACH motifs in different types of RNAs in Glioblastomas (GB) and Low-Grade Gliomas (LGG). **C** A radar plot display lncRNA m6 A modification levels in each patient studied. The orange and green zones represent glioblastoma and LGG patients, respectively. The blue dotted line indicates the overall m6 A modification level of lncRNAs in gliomas when considering all RRACHs, while the red dotted line shows the level when only RRACHs with a read coverage of 3 or more are included. The gray area in the radar plot illustrates the average percentage of lncRNAs with m6 A modifications detected in the GB (0.98%) and LGG groups (1.34%) (coverage ≥ 3). **D** A scatter boxplot comparing lncRNA methylated RRACHs in the LGG and GB groups

We identified 6,323–41,240 m6 A-methylated RNA molecules in different glioma patient samples. In addition, we identified 60–748 unique m6 A-modified lncRNAs for individual gliomas. Most of the modified adenosines were annotated to protein-coding RNA transcripts and covered 98.5% of all identified RRACH motifs (a specific RNA sequence motif where methylated adenines were analyzed), whereas RRACHs in lncRNAs accounted for only 1.16%, and those in pseudogenes and other biotypes accounted for less than 0.23%, as shown in Fig. 1A. The annotation of modified RRACH motifs was similar in both glioma groups: most of the alignments were to protein-coding transcripts: 98.18—99.2% in GBs and 97.95—98.85% in LGGs. In contrast, only 0.57–1.4% (GBs), and 0.93–1.69% (LGGs) of all detected RRACHs motifs were assigned to lncRNAs (see Fig. 1C).

We found that the overall m6 A modification levels of protein-coding, lncRNAs and pseudogene RNAs differed significantly between the GB and LGG groups (unpaired t-test, *p* < 0.01) and were higher in lower grade tumors (Fig. 1B). On average, 15.84% of all RRACHs within lncRNAs were modified in the GB group, while in the LGG group, adenine methylation in lncRNAs attained 23.73%, as shown in Fig. 1D.

**Fig. 2:**
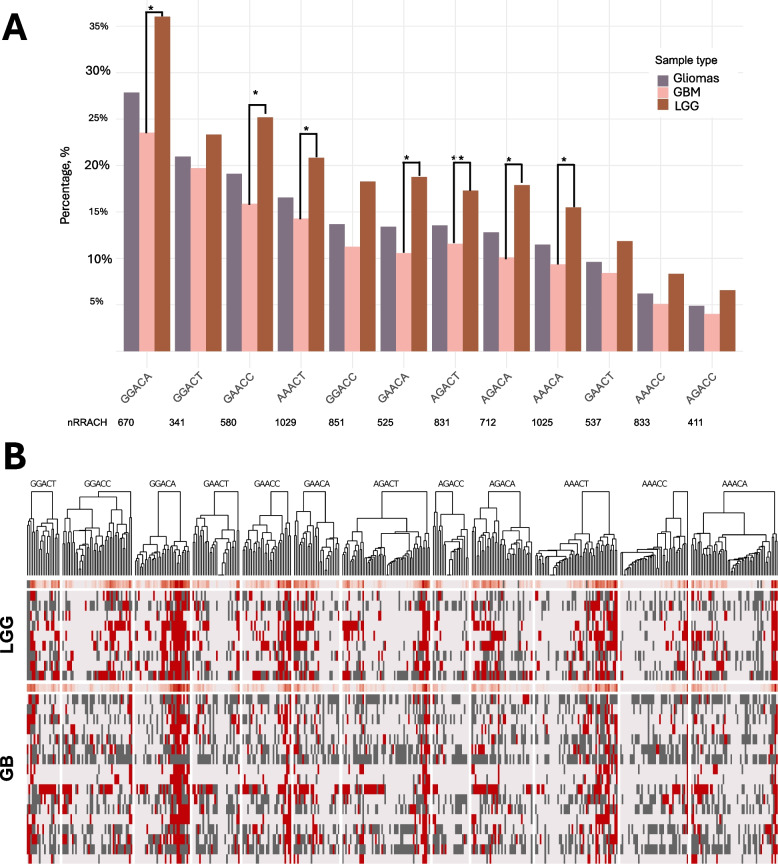
Overview of m6 A methylated RRACH motifs on lncRNAs in low grade glioma (LGG) and glioblastoma (GB) tissues. **A** The percentage (%) of RRACH motifs with detected m6 A in the LGG and GB groups. nRRACH depicts the total number of specific motifs detected in gliomas (*n* = 26). **B** The heat-map of m6 A hits sorted by motif (RRACH) type revealing m6 A methylation distribution over the different RRACHs

M6 A modifications in lncRNAs in gliomas were displayed predominantly in 12 RRACH motifs of which seven (GGACA, AGACT, GAACC, AAACT, AGACA, GAACA, and AAACA) were found to be significantly more frequently (up to 3.6-fold difference) m6 A methylated in LGGs as compared to GB specimens, see Fig. 2A. The motif GGACA showed notably higher methylation frequency (37.9%) compared to other RRACHs ranging from 6.8% (AGACC) to 37% (GGACA) in gliomas. However, in LGG the most frequently methylated RRACH was AGACT (60% of LGG cohort), while in GB – GGACA (33.4% of the cohort). We also noticed that RRACH motif m6 A modification frequency showed a tendency of moderate correlation with the total motif frequency in gliomas transcriptome (Pearson’s correlation, *r* = 0.57, *p* = 0.05).

### The lncRNAs m6 A profiling partitioned LGGs cohort while GBs stayed at one piece

**Fig. 3:**
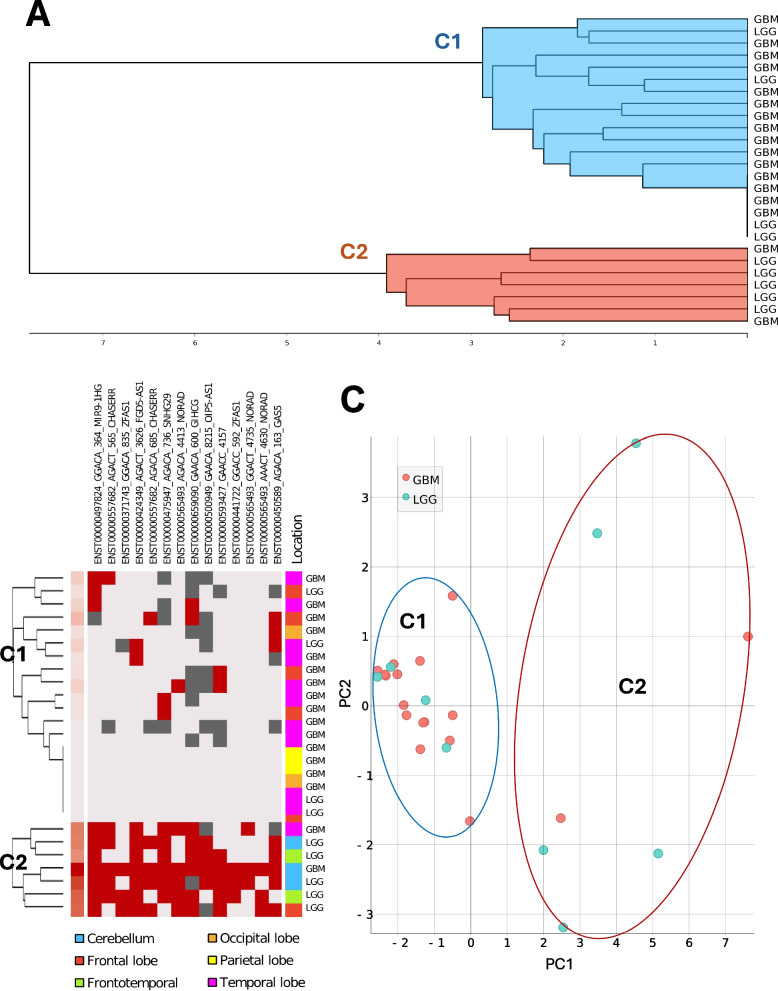
Gliomas clustering based on m6 A profile. **A** Hierarchical clustering dendrogram of the glioma m6 A modified lncRNA profile dataset, using adenine methylation status data from 14 lncRNA RRACHs. Glioma samples are grouped into C1and C2 clusters based on the similarity of m6 A pattern. **B** Heat-map of m6 A methylation profiles of RRACH motifs in 14 selected lncRNAs m6 A hits across different clusters. Red indicates methylated adenine (mod), light gray indicates unmethylated adenine (unmod), and dark gray indicates an unidentified adenine state within the RRACH motif. Tumor location of each case is shown on a color as follows: blue – Cerebellum, red – Frontal lobe, Green – Frontotemporal, orange – Occipital lobe, yellow – Parietal lobe, purple – Temporal lobe. **C** Principal component analysis (PCA) plot of glioma cases, displaying the first two components (explaining 64.6% of variance) generated using the 14 most informative RRACH motifs

To elucidate whether the m6 A profiles of lncRNAs differ between glioma specimens we performed agglomerative hierarchical clustering. The analysis incorporated 442 RRACH motifs (arranged within 42 lncRNA genes) that overlapped in more than 50% of the cohort samples analyzed. The following analysis encompassed m6 A status information only. The distances between samples were measured using Euclidean distance metrics. Ward´s linkage was applied to combine clusters via an agglomerative approach while k-Means clustering algorithm was used to identify reasonable number of the clusters (suppl. fig S2B). The samples were primarily clustered into 2 clusters (pC1 and pC2), of which pC2 mainly consisted of LGG cases (5/7) while pC1 was primarily consisted of GB cases (15/19) (suppl. Fig. S1). Following, meaningless m6 A sites (RRACHs that were similarly modified across the samples) were removed applying chi-square scoring for feature selection (target variable – assignment to cluster). The analysis led to the selection of 14 features (RRACHs) based on significance threshold of chi-square scores (*p* < 0.005, χ2 > 7.88, df = 1, see suppl. table St2). Next, hierarchical clustering analysis was performed utilizing 14 features—RRACH motifs (arranged within 10 lncRNA genes) applying the same parameters as indicated above. Gliomas were clustered again into 2 clusters—C1 and C2 (based on K-means scores), which compositions was the same as from primary clustering, see Fig. 3A. The consistency of clusters was evaluated by applying silhouette score analysis which revealed good to weak clustering structure for clusters (C1 and C2–0.503 and 0.181, respectively, see suppl. Fig. S3). Visualization of multidimensional scaling (MDS) confirmed silhouette analysis and showed closer distances between C1 samples than C2 samples based on 14 RRACH information, see suppl. fig. S4. Moreover, similar pairs test also confirmed this. To conclude, LGGs were partitioned between C1 and C2, while GB were consistently clustered together. To clarify the common features connecting samples in the clusters that were formed based on m6 A profile of lncRNAs we applied molecular, pathological and clinical features comparison based on cluster groups.

### Associations of the lncRNA m6 A profile with glioma patient clinical characteristics

Next, we checked if m6 A clusters C1 and C2 were associated with glioma patient clinical variables: patient age, tumor location, tumor molecular profile, and patient survival (suppl. fig. S5-6). Here we found that glioma median patient age (C1: 64.5 (± 17.4) vs C2: 47.7 (± 19.5), *p* > 0.05), tumor IDH status (IDHwt vs IDHmut, *p* > 0.05), molecular subtype (CL, MES, PN; *p* > 0.05), MGMT methylation status (Methylated vs unmethylated, *p* > 0.05) did not differ between lncRNA methylation clusters, suppl. Table St1. Similarly, patient age has no significant link with averaged m6 A level in different type of transcripts (*p* > 0.05). However, we noticed the tendency of cluster association with pathology grade (LGG vs GB, *p* = 0.054), while Ki-67 index expression (low vs. High, *p* = 0.04), and tumor location were found to be significantly associated with m6 A clusters (*p* < 0.01). Specifically, tumors located in brain cerebellum (*n* = 3) were highly m6 A modified on lncRNAs as compared to tumors in other locations (frontal lobe, *n* = 5, *p* = 0.003; frontotemporal lobe, *n* = 2, *p* = 0.08; occipital, *n* = 2, *p* = 0.038; parietal, *n* = 2, *p* = 0.007; temporal, *n* = 11, *p* < 0.001). While intracranial gliomas are common, cerebellar GB (c-GBM) are rare and make up for 0.4–3.4% of all adult GB cases with no significant difference in the survival time between patients with cerebellar and supratentorial GB (Takahashi et al. 2014; Grahovac et al. 2009). However, the difference appears in different types of cells which undergo malignant transformation depending on location in the brain. According to genomic profile c-GBMs may have originated from oligodendrocyte progenitor cells (Cho et al. 2019). Our study revealed the uniqueness of cerebellar tumors encompassing both LGG and GB in terms of increased m6 A levels in lncRNAs. The latter characteristic was able to cluster tumors of different malignancy to single cluster, likewise epigenetic DNA methylation pattern being specific and very stable in tumor regions reflecting the cell of origin in gliomas (Capper et al. 2018). However, more detailed associations between tumor localization and RNA modification should be investigated in a larger sample.

Next, we checked whether patient survival differed significantly between m6 A clusters. Kaplan–Meier analysis with the log-rank test revealed that the survival curve of the C2 cluster (which consisted of five LGGs and two GBs) showed the tendency to survive longer than that of the C1 (15 GB + 4LGGs) cluster (log-rank p = 0.17; df = 1; X^2^ = 1.88), suppl. fig. S7. Nevertheless, such the tendency is most likely predetermined by tumor grade than methylation of lncRNAs because Cox regression ranked 5 features from most to least important as follows: Ki-67, location, tumor grade, IDH status and cluster (should be noted that none of the analytes was significantly associated with survival risk). Therefore, the status of m6 A modifications had no significant value in predicting post-surgical survival time in our glioblastoma or low-grade glioma cohorts.

### Selected m6 A RRACHs on lncRNAs show high level of methylation in subpopulation of LGGs

Further analysis of individual m6 A motifs (RRACHs) revealed that the methylation level of almost all selected motifs in the GB group was 1.08 to 4.2-fold lower than that in the LGG group. However, among the lncRNAs that are differentially modified by m6 A the only *FGD5-AS1* passed chi-square test for difference in methylation proportions between pathology groups (*p* < 0.05, chi-square test), see Table 1. Our preliminary data suggests that transcriptome-wide demethylation contributes to tumor progression in gliomas, however low m6 A methylation in subpopulation of LGGs (4 out of 9) doesn’t fit into this hypothesis and should be clarified in further studies with more abundant cohort of low-grade glioma samples. Our preliminary data suggest that transcriptome-wide demethylation contributes to tumor progression in gliomas. However, the low m6 A methylation observed in a subset of LGGs (4 out of 9) does not align with this hypothesis and requires further investigation in a larger cohort of low-grade glioma samples.

**Table 1:** Differentially m6 A modified 14 RRACHs positions in 10 lncRNAs in GB & LGG, and C1 & C2

Gene name	Gene ID	Transcript ID	RRACH motif	RRACH Start	m6 A % GB (*n* = 17) vs LGG (*n* = 9)	*χ*2 test, *p* value	m6 A % C1 (*n* = 19) vs C2 (*n* = 7)	*χ*2 test, *p* value
*MIR91HG*	ENSG00000125462	ENST00000497824	*GGACA*	364	25.0 vs 66.7	0.08	16.7 vs 100	< 0.001
*CHASERR*	ENSG00000272888	ENST00000557682	*AGACT*	565	18.8 vs 33.3	0.63	5.6 vs 71.4	0.002
*ZFAS1*	ENSG00000177410	ENST00000371743	*GGACA*	*835*	5.9 vs 25.0	0.23	0 vs 42.9	0.015
*FGD5 AS1*	ENSG00000225733	ENST00000424349	*AGACT*	*3626*	17.6 vs 66.7	**0.03**	10.5 vs 100	< 0.001
*CHASERR*	ENSG00000272888	ENST00000557682	*AGACA*	685	12.5 vs 33.3	0.31	5.6 vs 57.1	0.012
*SNHG29*	ENSG00000175061	ENST00000475947	*AGACA*	736	30.8 vs 44.4	0.66	13.3 vs 85.7	0.002
*NORAD*	ENSG00000260032	ENST00000565493	*AGACA*	*4413*	17.6 vs 33.3	0.63	5.3 vs 71.4	0.002
*GIHCG*	ENSG00000257698	ENST00000659090	*GAACA*	600	36.4 vs 57.1	0.63	16.7 vs 100	0.002
	ENSG00000268205	ENST00000593427	*GAACC*	*4157*	20.0 vs 50.0	0.18	12.5 vs 71.4	0.011
*OIP5 AS1*	ENSG00000247556	ENST00000500949	*GAACA*	*8215*	10.0 vs 42.9	0.25	0 vs 80	0.002
*ZFAS1*	ENSG00000177410	ENST00000441722	*GGACC*	592	5.9 vs 22.2	0.27	0 vs 42.9	0.013
*NORAD*	ENSG00000260032	ENST00000565493	*GGACT*	*4735*	11.1 vs 11.8	1.00	0 vs 42.9	0.013
*NORAD*	ENSG00000260032	ENST00000565493	*AAACT*	4630	5.9 vs 22.2	0.27	0 vs 42.9	0.013
*GAS5*	ENSG00000234741	ENST00000450589	*AGACA*	163	21.4 vs 62.5	0.08	18.8 vs 83.3	0.011

Significant differences (*p* < 0.05) are indicated in bold

When comparing individual RRACHs between m6 A clusters C1 vs C2, chi-square test revealed all 14 RRACHs being differentially modified between clusters (*p* < 0.05), Table 1. Heatmap visualization between C1 and C2 supports individual RRACHs comparison and reveals the highest m6 A rates at selected RRACH motifs in the second cluster (mostly composed of LGG samples) (Fig. 3B).

Principal component analysis (PCA) of the 14 RRACH motifs, was performed to visualize spatial differences and similarities among clusters. The first two components of the PCA explained 64.6% of total variance. Low methylation cluster C1 points (15 GB and 4 LGGs) showed smaller distribution and was located closer to the center of cluster convergence, compared to highly methylated C2 mainly composed of LGG indicating relevant differences in m6 A profiles of clusters, see Fig. 3C. These results suggest that differential epitranscriptomic m6 A profiles exist in different glioma types and likely represent subgroups of tumors.

### Major m6 A-modified lncRNAs are highly expressed in LGG

**Fig. 4:**
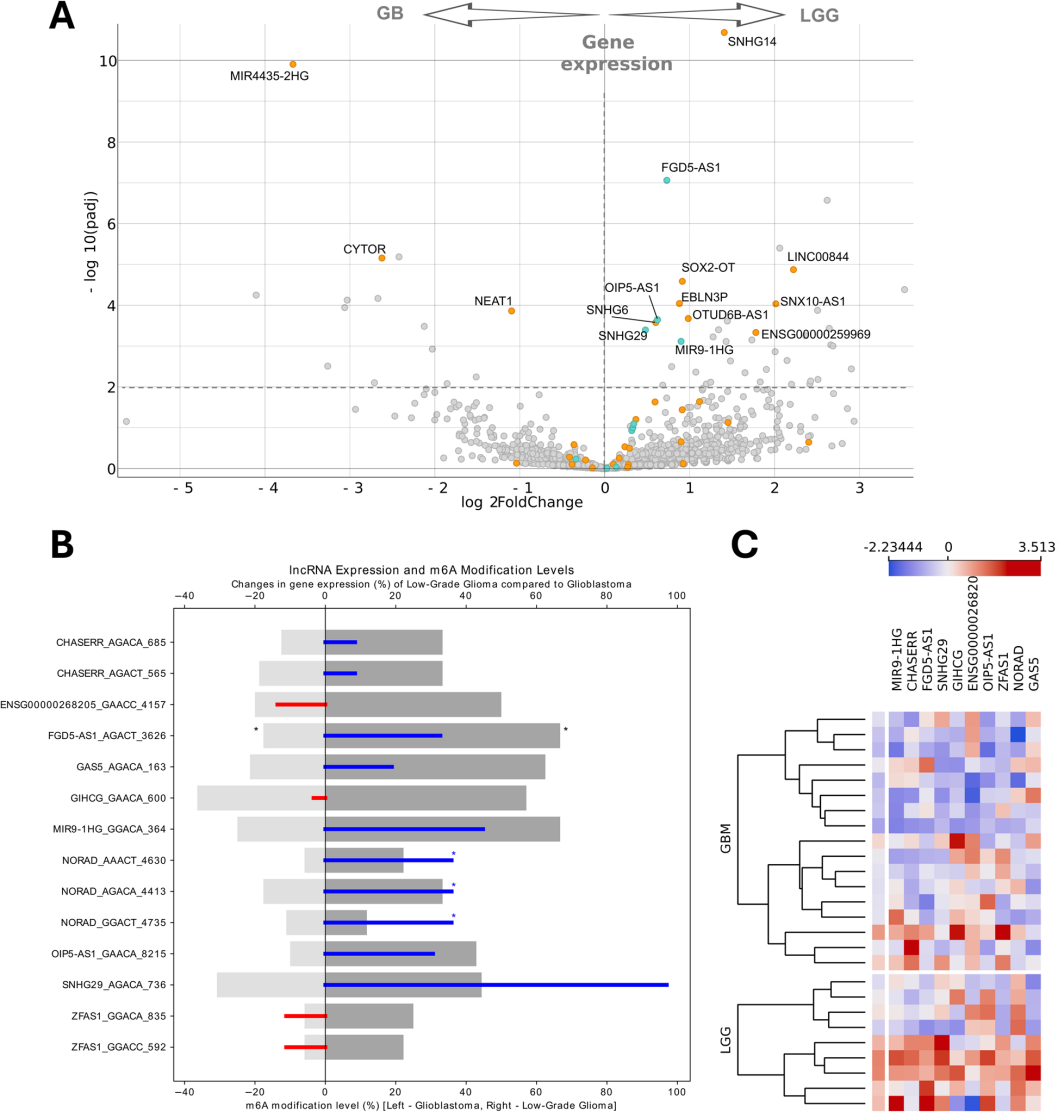
m6 A modified lncRNA expression patterns in gliomas. **A** Volcano plot of differentially expressed lncRNAs between GB and LGG. Dots on the left represent higher lncRNA expression in GB, while dots on the right represent higher expression in LGGs. Blue and orange dots indicate lncRNAs with informative RRACH motifs used for clustering gliomas into three clusters based on 442 RRACHs. Blue dots denote lncRNAs that were selected as 10 the most informative features for clustering gliomas into two clusters. **B** Comparison of lncRNA m6 A modification (grey bars) and expression (blue and red bars) in the GB and LGG groups for the 14 selected RRACHs. Scale from 0 to 100 indicates increasing m6 A and/or expression level in LGGs, scale from 0 to −100 indicates increasing m6 A and/or expression level in GB. Asterisk indicates significant (*p* < 0.05) increase of methylation or expression in LGG or GB, accordingly. **C** The heat-map of the expression of selected 10 lncRNAs (containing 14 RRACH motifs) subdivided according to the pathological grade

Next, we performed a DEG analysis (DEseq2) comparing GB and LGGs to evaluate whether differentially m6 A modified lncRNAs between cluster are expressed equally in both malignancies. A total of 442 previously selected RRACHs were distributed among 42 lncRNA genes (66 transcripts), of which 15 genes were found to pass the deseq2 adjusted *p*-value threshold (*p* < 0.05), Fig. 4A. Analysis revealed significantly increased expression of MIR4435-2HG, CYTOR, and NEAT1 in GB specimens compared to LGG specimens. The expression of twelve lncRNAs (SNHG14, FGD5-AS1, LINC00844, SOX2-OT, EBLN3P, OTUD6B-AS1, ENSG00000259969, OIP5-AS1, SNHG6, SNHG29, MIR9-1HG, SNX10-AS1)) was significantly higher in LGGs than in GB (Fig. 4A).

Further we checked if the expression of selected lncRNAs (10 genes) differ between malignancy grade (GB vs LGG) of the glioma. Analysis revealed that MIR9-1HG, FGD5-AS1, SNHG29, OIP5-AS1 and NORAD levels were significantly higher in LGGs as compared to GBs while the rest five lncRNAs expression was evenly distributed across the groups, see suppl. fig. S8. Similarly, the expression of selected lncRNAs was significantly higher in high methylation – C2 cluster, see suppl. fig. S9, S7B. Next, we asked if m6 A methylation is limited by the abundance of RNA levels in selected m6 A hits (14 RRACHs). LncRNA m6 A methylation and expression association analysis (TPM values used) showed that methylation at MIR9-1HG (Point-Biserial Correlation *r* = 0.439, *p* = 0.028) and ZFAS1 (Point-Biserial Correlation *r* = 0.609, *p* = 0.0012 (GGACA); *r* = 0.396, *p* = 0.045 (GGACC)) was significantly associated with gene expression, see suppl. fig. S10. The methylation in both genes was associated with an increased abundance of RNA levels. Moreover, the trend of higher lncRNA expression in m6 A methylation group was observed for the majority (7/10) of lncRNAs analyzed, see suppl. fig. S10. This might be explained via multisite cumulative effect of m6 A modification when several nearby m6 A hits might have a faster/higher trigger effect than single m6 A. Current analysis was limited to single m6 A site comparisons since there is no justified method to combine m6 A sites information of the same transcript/transcript site up to date. Therefore, the combination of RRACH status information might provide an additional layer of information regarding lncRNAs methylation and expression connection.

To summarize, the expression of half (5/10) of the selected lncRNAs differ significantly, while the remaining lncRNAs were equally expressed between LGG and GB, despite all of them being highly methylated in LGGs (Fig. 3B). However, whether lncRNA downregulation occurs in conjunction with transcript demethylation during tumor progression remains to be determined. Our data demonstrates that lncRNAs exhibiting differential m6 A modifications between LGG and GB tissues do not necessarily differentiate tumors at the gene expression level.

### Gene expression of the m6a machinery does not reflect the global m6 A modification level in gliomas

The robust modulation of m6 A effectors (writers and erasers) in cell lines results in altered m6 A levels (Cui et al. 2017; Vu et al. 2017; Li et al. 2017). To check m6 A machinery gene activity association with m6 A level in our cohort we applied Pearson correlation analysis which revealed no association between expression of m6 A writers (METTL3, METTL5, METTL14, METTL16, WTAP, VIRMA, and ZCCHC4) and erasers (FTO and ALKBH5) and the m6 A methylation levels of RNAs (lncRNAs, protein-coding mRNAs, pseudogene RNAs, etc.) in the whole cohort (*p* > 0.05); see suppl. table St3. It is worth mentioning that the difference in the expression of m6 A effectors between GB and LGG tumors in our cohort aligned with the TCGA data (suppl. fig. S11-15). Similarly, as in the TCGA dataset, WTAP expression was significantly greater in GB specimens than in LGGs (*p* = 0.004), while FTO mRNA levels were lower in GB than in LGG specimens (*p* = 0.051). In the case of the METTL3, METTL14, and ALKBH5 m6 A effectors, we found similar gene expression shift tendencies in our cohort as compared to those in the TCGA dataset (suppl. fig. S11-15).

### Differences in expression of protein-coding genes between C1 and C2 samples revealed genes involved in immune processes

Clustering analysis based on m6 A status of lncRNAs identified two groups of glioma samples, thus following differentially expressed genes (DEG) analysis was performed to identify C1 and C2 groups specific mRNA genes to find common functional variables. DEG revealed 10 genes (BST2, C1QB, CD14, FCGR3 A, HLA-DPA1, HLA-DRA, LGALS3, S100 A11, SPP1, SRGN) significantly upregulated (*p* < 0.01; FC > 1.5) in cluster C1 and 2 genes (YPEL3, RPL26P19) in C2, see suppl. fig. S16 A. Following, gene ontology (GO) analysis for biological processes (*p*-value cutoff < 0.0001) was performed using C1 cluster specific genes that resulted in 11 GO terms (see suppl. table St4, fig. S17). Most of the processes were related to immune activities terms. It is known that strong correlation exists between the immune system processes and glioma development and prognosis (Jayaram and Phillips 2024). GO analysis showed genes which influence tumor microenvironment by modulating the activity of immune cells like macrophages (S100 A11, FCGR3 A, C1QB), microglia, and T cells, as well as promoting inflammatory signaling (SRGN) that supports tumor growth, invasiveness, and resistance to therapies (Wang et al. 2021; Feng et al. 2024; Manou et al. 2024). In conclusion, the genes C1QB, S100 A11, FCGR3 A, SRGN, HLA-DRA, CD14, LGALS3, BST2, SPP1, and HLA-DPA1 play significant roles in shaping the immune landscape of GB, contributing to both immune activation and immune evasion. Further we evaluated if the immune system processes associated genes expression are homogeneously upregulated in C1 cohort across the samples encompassing GB and LGGs. Heat map visualization of selected genes (TMP values) revealed that upregulated immune system processes associated genes levels in C1 were primarily predetermined by GB cohort while expression in LGGs from both clusters were uniformly low, see suppl. fig. S15B. Thus, m6 A methylation differences between clusters cannot be explained via processes related to cells immune system response.

### LGG cohort dispersed across C1 and C2

The DEG analysis between C1 and C2 identified immune system response genes mainly related to GB cohort rather than clusters. Thus, next only LGGs samples were analyzed based on cluster information. DEG analysis of C1 (*n* = 4) and C2 (*n* = 5) LGGs resulted in 11 genes (CCK, CHN1, IFI44L, IFIT1, ISG15, NEFL, NRGN, SELL, SYT1, SNAP25) significantly upregulated (*p* < 0.05; FC > 1.5) in C1 cluster and 3 genes (OLIG1, SEZ6L, ESNG00000185641) upregulated in C2 cluster, see suppl. fig. S18. The GO-term analysis of differentially co-expressed genes for biological processes using list of 11 genes (*p*-value cutoff < 0.0001) revealed 8 terms generally related to neuron development, suppl. table St5, fig. S19. Neuron development associated genes might indicate healthy or/and well differentiated tissue impurities in samples with higher expression of these genes. However, it may also indicate that m6 A methylation in low-grade gliomas may contribute to the pathogenesis primarily by affecting genes active in neural development. It was proposed that the most differentiated cells in the CNS upon defined genetic alterations undergo dedifferentiation to generate a NSC or progenitor state to initiate and maintain the tumor progression, as well as to give rise to the heterogeneous populations observed in malignant gliomas (Venteicher et al. 2017). In our study, the majority of DEGs between LGGs are associated with normal neuronal development, like CHN1 and NEFL (NFL) are expressed in cerebral cortex and active in synaptic genesis and function (Volk et al. 2010), while in cancer those genes play as oncogenes (Zhao et al. 2021). For example, overexpression of CHN1 gene in cervical cancer was associated with accelerated cancer cell invasion, migration and tumor progression (Zhao et al. 2021). Overexpression of neuronal development genes in C1 LGGs supports the hypothesis that more aggressive LGGs may exhibit an m6 A pattern similar to that of GBs.

Further, transcriptomic and antibody-based mapping analysis by Sjostedt et al. listed CCK and NRGN genes to be highly enriched in the human brain cerebrum while very weakly expressed in brain cerebellum (Sjöstedt et al. 2020). Interestingly, our DEG analysis also defined CCK and NRGN gene expression differences between C1 and C2 LGG subgroups as expected with the weaker expression in cerebellar low-grade gliomas (C2). So, here we hypothesize that LGG clustering and m6 A methylation pattern could also represent tumor location entities.

To conclude, here we agree with other authors that clinicopathological assessment, valuable to distinguish distinct grades of glioma, does not address genetic and biologically different subgroups that are nested within each grade category (Chang et al. 2016).

## Discussion

Our study reports epitranscriptome-wide posttranscriptional m6 A modifications on lncRNAs across multiple primary human glioma tissues and their integration with gene expression, disease stage, molecular markers, tumor size, location and patient survival. Here, we defined transcriptome-wide m6 A differences in lncRNAs between 9 low-grade gliomas and 17 high-grade gliomas, revealing decreased methylation levels in malignant glioblastomas. A total of 442 RRACH motifs, spanning 42 lncRNAs, stratified gliomas in two clusters, while 14 of RRACHs (10 lncRNAs) were defined to differentiate between LGG and GB, and differentially methylated glioma clusters C1 and C2.

Here, for the first time, we found that higher malignancy gliomas contain fewer m6 A modifications in noncoding and protein-coding RNAs. Additionally, lncRNA m6 A profile distinguished differentially methylated low-grade gliomas that dispersed between clusters. This distribution of LGG among methylation clusters may be partly explained by the differences in tumor localization. Open-access Brain Atlas resource recently published regional gene expression differences in brain, showing Cerebellum has the most regionally enriched genes (Sjöstedt et al. 2020). Our study revealed the uniqueness of cerebellar tumors encompassing both LGG and GB in terms of increased m6 A levels in lncRNAs. The latter characteristic was able to cluster tumors of different malignancy to single cluster, likewise epigenetic DNA methylation pattern being specific and very stable in tumor regions reflecting the cell of origin in gliomas (Capper et al. 2018). It is worth mentioning that the relatively small sample size in our study may limit the generalizability of our results to larger cohorts. Therefore, future research with large glioma cohorts is warranted to corroborate these findings.

Next, our findings confirm recent studies showing that reduced m6 A likely promotes tumorigenesis. Reduced m6 A levels in ADAM19 mRNA (Cui et al. 2017) and in FOXM1 (Zhang et al. 2017) promote glioma stem cell proliferation and self‐renewal and ultimately lead to tumorigenesis. In approximately 70% of endometrial tumors, decreased m6 A levels result in AKT activation and enhanced proliferation and tumorigenicity (Liu et al. 2018). Reduced levels of m6 A modification in breast cancer stabilize KLF4 and NANOG mRNA, leading to the enrichment of stem cells and promoting tumorigenesis (Zhang et al. 2016a, 2016b). This highlights the potential role of decreased RNA methylation in tumor development and progression. Likewise, the opposite role of m6 A has also been reported. For instance, in acute myeloid leukemia (AML), increased m6 A levels on SP1, MYB, MYC, BCL2, and PTEN enhance the stability and translation of their corresponding mRNAs, contributing to the onset and progression of AML. (Vu et al. 2017; Weng et al. 2018; Barbieri et al. 2017). Similarly, in hepatocellular carcinoma, increased m6 A modification of SOCS2 speeds up its degradation, thereby promoting tumor progression (Chen et al. 2018). In breast cancer, increased m6 A methylation of HBXIP accelerates tumorigenesis (Cai et al. 2018). Therefore, RNA methylation triggers diverse outcomes in cancer-related biological functions depending on the target of the modification. However, it should be noted that all of these studies focused on quantifying m6 A on mRNA molecules, and none focused on noncoding lncRNAs. Importantly, compared to other known studies in literature, we present a new approach that uses native RNA sequencing to identify and quantify m6 A modifications present in innate RNA molecules in glioma tumor tissue.

Furthermore, our data shed light on the glioma lncRNA epitranscriptome, which has not been previously characterized. Here, using long-read dRNA-seq we identified 10 differentially m6 A modified lncRNAs (*GAS5, MIR9-1HG, GIHCG, CHASERR, FGD5-AS1, ZFAS1, SNHG29, NORAD, OIP5-AS1*, and one novel gene) in different glioma malignancies and methylation clusters.

Specific lncRNAs like *MiR9-1HG (C1Orf61)*, *CHASSER*, *ZFAS1* were highly methylated (≥ 50%, 1–2 significant RRACHs) in our glioma set. Upregulated expression of *MIR9-1HG* gene was reported in glioma stem cells as compared to differentiated tumor cells (Wang et al. 2018). Interestingly, our previous m6 A methylation study on GSC NCH421k and U87MG showed highly modified *MiR9-1HG* in GSCs (data not shown) (Steponaitis et al. 2022), Dominissini et al. (2012) reported no methylation of the latter gene in normal brain tissue (Dominissini et al. 2012).

CHASERR (*LINC01578)* are almost completely uncharacterized lncRNA found upstream of and transcribed from the same strand as *Chd2* helicase and seems to participate in cis regulation of this gene. *Chd2* is associated with chromatine remodeling (Rom et al. 2019). LINC01578 drives colon cancer metastasis and correlates with patient poor prognosis (Liu et al. 2020b). As to our knowledge we are the first here to report on *CHASSERR* m6 A modification in gliomas.

*ZFAS1* gene expression downregulation was found to be associated with progressing gliomas and worse overall survival (Matjašič et al. 2017). Here we found that *ZFAS1* and *MIR9-1HG* m6 A were associated with increased abundance of RNA level. Lower *ZFAS1* m6 A modification in GB could contribute to lower lncRNA expression and progression of tumors. Further, *ZFAS1* was shown to be m6 A methylated in glioma stem cells NCH421k (Steponaitis et al. 2022), which drives gliomagenesis. As to our knowledge up to date there is no data on lncRNAs epitranscriptomic landscape in gliomas which could support or conflict our findings. Our findings provide an overview of m6 A-modified lncRNAs that are highly modulated in gliomas and may serve as potential targets for functional studies and therapeutic modulation. To establish their biological relevance, future research involving m6 A site-directed mutagenesis or gain/loss-of-function approaches such as CRISPR-Cas-based strategies or manipulation of m6 A regulators will be essential to elucidate their role in tumor progression. Furthermore, these selective m6 A-modified lncRNAs may offer opportunities to enhance the efficacy of current immunotherapies used in glioma treatment (Sharma et al. 2025).

Our preliminary data in glioma tissues suggests that transcriptome-wide demethylation partially contributes to tumor progression in gliomas. However low m6 A methylation in subpopulation of LGGs (4 out of 9) doesn’t fit into this hypothesis unless this could be associated with regional methylation differences in the brain. This should be confirmed in future studies.

At the non-coding RNA transcript level, several studies have shown that m6 A appears to be linked to specific aspects of RNA stability and processing (Jacob et al. 2017; Thapar et al. 2019). Visvanathan and colleagues demonstrated that the majority of noncoding lincRNAs were upregulated (opposite to what was observed for mRNAs) under induced depletion of METTL3 in glioma stem cells. However, the expression of the lincRNAs XIST, MALAT1 and H19, which are m6 A-modified transcripts, increased in glioma stem cells (Visvanathan et al. 2019). Recently, the Wang group (Chang et al. 2021) demonstrated that METTL3 promoted the malignant progression of IDH-wt gliomas while upregulating the expression of the lncRNA MALAT1 by enhancing its stability via m6 A modification.

Our results are in agreement with these above-mentioned studies, as we also revealed an increased expression of most of lncRNAs in the highly methylated cluster (C2) compared to the low methylation cluster (C1). However, we could not reiterate this at the individual lncRNA molecule level. Undeniably, multiple studies have also suggested that m6 A modifications function as regulators of cancer pathogenesis, but how they act in a lncRNA-dependent manner during glioma progression is unclear. The m6 A machinery can maintain the progression of tumors by modifying specific lncRNA molecules that regulate gene expression through certain pathways. Studies have shown that m6 A modifications might affect the localization and activity of lncRNAs, for instance, certain m6 A modifications can lead to the structural changes in MALAT1 (Zhou et al. 2016), which might regulate interactions between lncRNAs and m6 A-binding proteins, likely contributing to the aggressive progression of tumors (Ma et al. 2019).

The m6 A profiling of the glioma cohort revealed two distinct clusters, with differential gene expression analysis indicating enrichment of immune system–related genes in GB cluster. Given the well-established link between immune system activity and glioma development and prognosis, it is plausible that these m6 A-defined clusters represent differential immune microenvironment landscapes. Moreover, immune system-associated genes were uniformly low expressed in LGGs from both clusters, suggesting that LGGs represent a less inflammatory phenotype. Supporting this notion, studies in colorectal cancer have shown that immune cells from patients exhibit significantly higher m6 A modification levels compared to those from healthy individuals (Xie et al. 2021). This suggests that m6 A modifications may play a regulatory role in immune cell function within the tumor microenvironment, a phenomenon that can be inferred from bulk tissue m6 A profiling.

m6 A methylation in low-grade gliomas may contribute to tumor pathogenesis predominantly by modulating genes involved in neural development (Yen and Chen 2021). The overexpression of neurodevelopmental genes observed in Cluster 1 LGGs supports the hypothesis that more aggressive, thus less differentiated LGGs may acquire an m6 A modification profile resembling that of GBs. Alternatively, the m6 A landscape in LGGs may be shaped by immune system components, which could also correlate with increased tumor aggressiveness and poorer clinical outcomes (Cao et al. 2023). Nonetheless, single-cell m6 A profiling would provide a more granular understanding of the proposed m6 A–immune–differentiation axis in glioma progression.

Previous studies have shown that components of m6 A machinery (m6 A writers, erasers, and readers) are linked to tumorigenesis in glioblastoma. Associations between glioma malignancy grade and the expression of m6 A writers (METTL3, METTL14, WTAP, and RBM15), the reader YTHDF, and erasers (ALKBH5 and FTO) (Dong and Cui 2020; Chai et al. 2019; Wang et al. 2021) are shown. However, data on the relationship between the activity of m6 A machinery genes and RNA m6 A methylation levels remain limited. While most studies focus on the altered expression of m6 A machinery components, these changes do not necessarily correlate with abnormal m6 A levels, as demonstrated in the present study.

Many in vitro studies have shown that external manipulations of m6 A modulator expression can regulate m6 A levels (Sun et al. 2019). However, there is significant data gap regarding whether natural m6 A levels are closely linked to the abundance of the m6 A machinery. Most importantly, our findings revealed no correlation between the expression of known m6 A modulators and global RNA m6 A methylation levels in low- and high-grade gliomas. Given that the outcome of m6 A methylation can vary depending on the type of molecule and the location of the modification, the overall abundance of m6 A sites alone may not provide a comprehensive explanation. Furthermore, it cannot be excluded that m6 A modulators are dynamically controlled by different systems (Zhou et al. 2015; Wang et al. 2022), most of which are likely still undiscovered since the epitranscriptome research era is still in its infancy.

Also, post-transcriptional mechanisms, such as mRNA stability, translation efficiency, and protein degradation, can influence the actual levels and activity of m6 A modulators. For instance, the METTL3-METTL14 complex, responsible for m6 A methylation, requires proper assembly and localization to function effectively, while disruptions in these processes can lead to altered m6 A levels independent of mRNA expression (Lin et al. 2022). The bulk tissue sequencing may also mask cell-type-specific correlations between m6 A levels and modulator expression, especially when investigating cancerous tissues that are associated with varying tumor microenvironment characteristics and immune cell infiltration levels (Cao et al. 2023; Han et al. 2022). Liu et al. showed that in case of hepatocellular carcinoma, distinct m6 A modification patterns have been linked to different prognoses and tumor microenvironment landscapes, indicating complex regulation beyond modulator expression levels (Liu et al. 2022). To further elucidate the N6-methyladenosine modulator axis and its underlying interactions, single-cell analyses and proteomic studies should be employed to gain more precise insights into m6 A regulation in cancer.

Overall, we showed that m6 A modifications are targeted at lncRNAs in gliomas and likely promote glioma progression. Additionally, owing to the subtypes we demonstrate using m6a-lncRNA axis, we propose that m6a-associated lncRNAs may constitute a source of tumor heterogeneity. It is also worth mentioning some limitations of this study, 1) our study is confined to a relatively small number of glioma patients which may restrict the statistical power of our findings. 2) we primarily focused on poly-A marked lncRNA m6 A signals. More information on the non-poly-A transcripts might also be equally relevant. 3) Since mapping and quantifying RNA modifications in a full-length sequenced molecule is very challenging due to the high propensity for RNA breaks, some bias towards the 3’ end sites is to be expected in the output of dRNA-seq data. Consequently, due to the 3’-end bias and RNA fragmentation, we do not recognize all possible m6 As equally—m6 As at the 5’-end are recognized less frequently, or some are not recognized at all. 4) We used bulk tissue specimens, and single-cell m6 A profiling could provide cell-specific m6 A information thereby paving the way for targeted therapy. 4) The current study included m6 A analysis of RRACH motifs, while non-RRACH m6 A sites in the future also require equal attention. Nevertheless, our study provides new insights into the altered m6 A modifications of lncRNAs in gliomas and provides a basis for further exploration of this noncoding epitranscriptome.

## Supplementary Information

Supplementary Material 1.

## Data Availability

All data presented in the manuscript are deposited in public repository GEO under the accession number GSE282642 and can be reached by reviewers under the private token.
