# Supplementary material for: m6A-lncRNA landscape highlights reduced levels of m6A modification in glioblastoma as compared to low-grade glioma
Source: Mol Med. 2025 May 17;31:195. doi: 10.1186/s10020-025-01254-x (PMC12085027; doi:10.1186/s10020-025-01254-x)

Table St1. Clinical and phenotypical characteristics of glioma data sets: LGG vs GBM, and C1 vs C2. P-values were calculated were appropriate. Medians were compared using nonparametric independent samples Fisher's exact test, while categorical variables distribution between groups was tested by Pearson chi-square test. CL (classical), PN (proneural), MES (mesenchymal).

		**LGG**	**GBM**	*p-value*	**C1**	**C2**	*p-value*
Number of patients	26	9	17	-	19	7	-
Age median (min to max)	61.6 (24.2-85.4)	33.4 (24.2-71.6)	67.8 (50.1-85.4)	0.09	64.5 (24.2-85.4)	47.7 (26.9-78.0)	0.38
Gender	Male	3	7	0.69	7	3	0.77
	Female	6	10		12	4	
Tumor size median (min to max)		116.6 (38.5-171)	50.4 (7.3-293.2)	0.41	96.8 (7.3-293.2)	60.5 (38.5-247.5)	0.96
Tumor location	Cerebellum	2	1	0.1	-	3	**0.004**
	Frontal lobe	3	3		5	1	
	Frontotemporal	2	-		-	2	
	Occipital lobe	-	2		2	-	
	Parietal lobe	-	2		2	-	
	Temporal lobe	2	9		10	1	
Tumor subtype	CL	-	9	-	8	1	0.09
	PN	-	4		4	-	
	MES	-	4		3	1	
MGMT methylated	Yes	6	8	0.43	10	4	1
	No	3	9		9	3	
IDH1 mutated	Yes	8	0	**0.001**	4	4	0.15
	No	1	17		15	3	
Ki-67	Low	9	3	**0.001**	1	6	**0.03**
	High	-	14		18	1	
Overall median (min to max) survival (days)		3093 (1192-3829)	290 (32-2215)	**<0.001**	426 (32-3829)	1256 (253-3537)	0.07

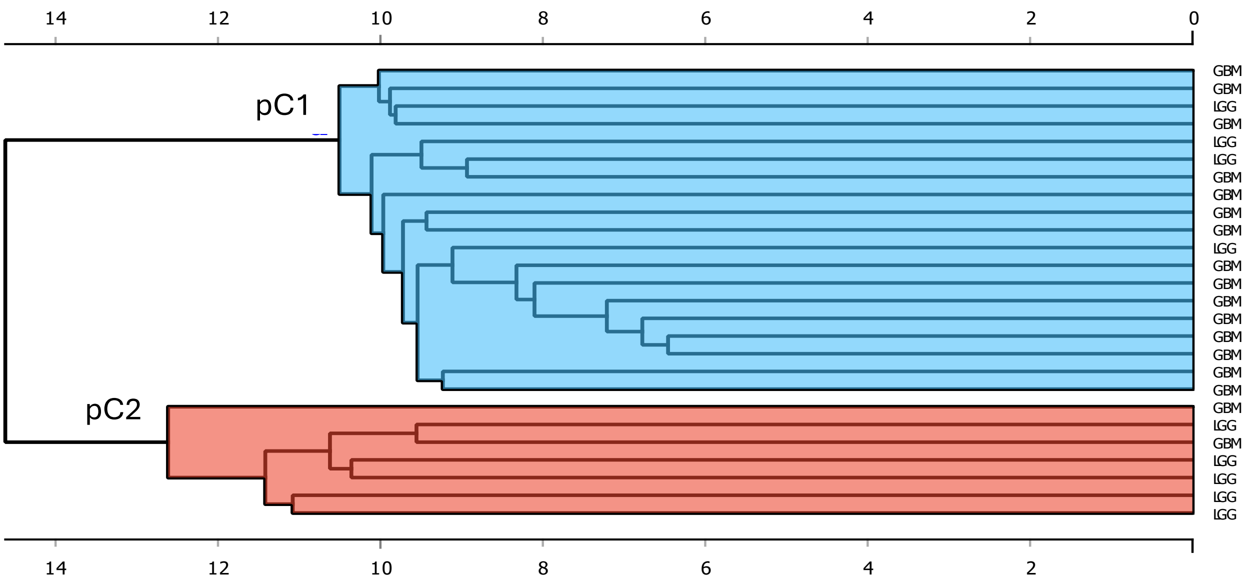

Fig. S1. Hierarchical clustering dendrogram of the glioma m6A modified lncRNA profile dataset, using adenine methylation status data from 442 lncRNA RRACHs.

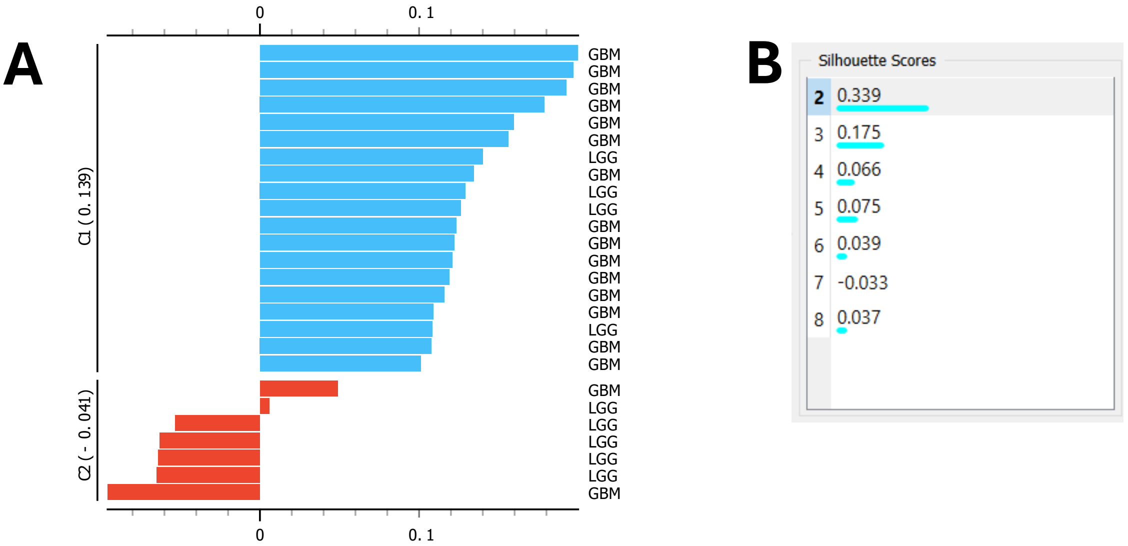

Fig. S2. (**A**) Silhouette analysis of clustering applying 442 RRACH information. (**B**) k-Means clustering analysis Silhouette scores for different numbers of clusters. The analysis showed optimal number of clusters.

Table St2. Features (RRACHs) table after feature selection (ranking) applying chi-square (χ^2) scoring. Colors indicates top lncRNAs RRACHs based on p-value threshold (green p<0,005; white – p<0,01; grey – p>0,05)

No	Feature	χ^2	p-value
1	ENST00000497824_GGACA_364_MIR9-1HG	16,8596	0,00004
2	ENST00000557682_AGACT_565_CHASERR	16,5188	0,000048
3	ENST00000371743_GGACA_835_ZFAS1	12,2975	0,000454
4	ENST00000424349_AGACT_3626_FGD5-AS1	11,8304	0,000583
5	ENST00000557682_AGACA_685_CHASERR	11,73	0,000615
6	ENST00000475947_AGACA_736_SNHG29	11,1218	0,000853
7	ENST00000565493_AGACA_4413_NORAD	9,70426	0,001838
8	ENST00000659090_GAACA_600_GIHCG	9,05388	0,002621
9	ENST00000593427_GAACC_4157_ENSG00000268205	8,79301	0,003024
10	ENST00000500949_GAACA_8215_OIP5-AS1	8,79301	0,003024
11	ENST00000441722_GGACC_592_ZFAS1	8,14286	0,004323
12	ENST00000565493_GGACT_4735_NORAD	8,14286	0,004323
13	ENST00000565493_AAACT_4630_NORAD	8,14286	0,004323
14	ENST00000450589_AGACA_163_GAS5	8,01353	0,004643
15	ENST00000450589_GGACT_356_GAS5	7,53589	0,006048
16	ENST00000557682_AGACA_867_CHASERR	7,22556	0,007187
17	ENST00000557682_GAACC_643_CHASERR	7,22556	0,007187
18	ENST00000411630_GAACC_178_DANCR	7,22556	0,007187
19	ENST00000411630_GGACA_536_DANCR	7,22556	0,007187
20	ENST00000620266_AAACT_2755_ENSG00000278730	7,22556	0,007187
21	ENST00000371743_AGACA_615_ZFAS1	7,1594	0,007457
22	ENST00000557682_GGACC_474_CHASERR	7,1594	0,007457
23	ENST00000581621_GGACT_2018_ENSG00000264772	7,04726	0,007939
24	ENST00000465270_AAACC_300_MIR9-1HG	7,04726	0,007939
25	ENST00000430245_GGACT_447_GAS5	6,99436	0,008177
26	ENST00000499521_GAACT_8468_ENSG00000230551	6,99436	0,008177
27	ENST00000602520_GAACA_96_SNHG8	6,49839	0,010797
28	ENST00000554133_AGACA_1531_CHASERR	6,49839	0,010797
29	ENST00000557682_GGACC_388_CHASERR	6,38267	0,011524
30	ENST00000310027_GGACC_492_MIR9-1HG	5,84918	0,015584
31	ENST00000657986_AAACC_3505_SOX2-OT	5,84918	0,015584
32	ENST00000581621_AAACA_2185_ENSG00000264772	5,5609	0,018366
33	ENST00000563192_AAACC_255_SNHG19	5,5609	0,018366
34	ENST00000441722_AGACT_887_ZFAS1	5,42857	0,01981
35	ENST00000565493_GGACA_4694_NORAD	5,42857	0,01981
36	ENST00000653276_GGACT_119_SNHG7	5,41353	0,01998
37	ENST00000371743_GAACC_676_ZFAS1	5,31779	0,021109
38	ENST00000501122_GAACT_3644_NEAT1	5,14662	0,023292
39	ENST00000653276_GGACC_529_SNHG7	5,10788	0,023817
40	ENST00000602520_AGACT_126_SNHG8	4,81704	0,02818
41	ENST00000581621_GGACC_2045_ENSG00000264772	4,78947	0,028634
42	ENST00000513560_GAACC_3922_NNT-AS1	4,78947	0,028634
43	ENST00000505030_AAACA_3583_LINC00461	4,57179	0,032503
44	ENST00000417721_AGACA_615_ZFAS1	4,57179	0,032503
45	ENST00000500949_AGACT_8093_OIP5-AS1	4,57179	0,032503
46	ENST00000591501_AAACC_1889_ILF3-DT	4,57179	0,032503
47	ENST00000651844_AGACA_7646_COPG2IT1	4,53067	0,033293
48	ENST00000653163_AGACA_846_CHASERR	4,33083	0,037428
49	ENST00000371743_GGACC_592_ZFAS1	4,26589	0,038885
50	ENST00000557682_AAACT_650_CHASERR	4,26589	0,038885
51	ENST00000475947_GGACT_839_SNHG29	4,26589	0,038885
52	ENST00000623091_GGACA_1603_HEIH	4,26589	0,038885
53	ENST00000593427_GAACA_3674_ENSG00000268205	4,0368	0,044518
54	ENST00000656289_GAACT_310_ENSG00000225792	4,0368	0,044518
55	ENST00000581621_AGACA_1864_ENSG00000264772	3,84005	0,050042
56	ENST00000610119_GAACA_384_ENSG00000272755	3,84005	0,050042

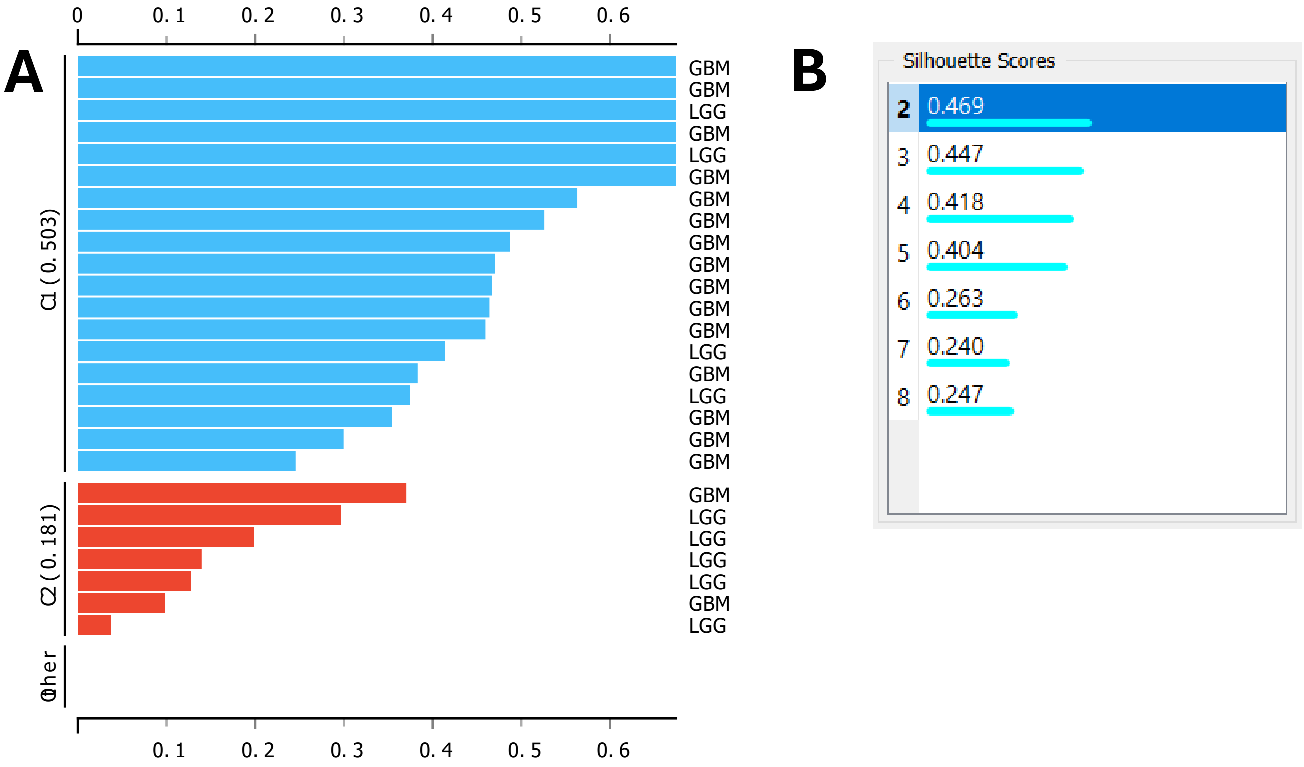

Fig. S3. (**A**) Silhouette analysis of clusters applying Euclidean metrics (used for 14 selected RRACH information). (**B**) k-Means clustering analysis: Silhouette score evaluation determined that the optimal number of clusters is 2, indicating a well-defined clustering structure for 2 clusters.

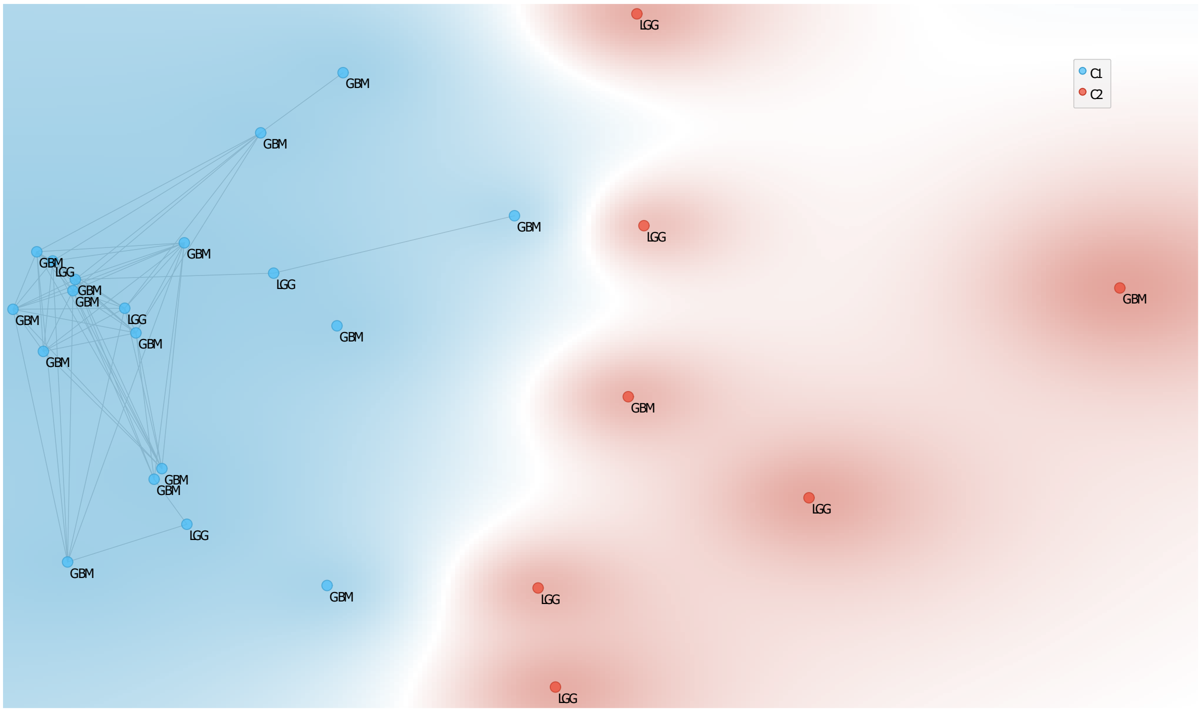

Fig. S4. Two clusters representing samples shown in multidimensional scaling (MDS) plot revealed closer distances between cluster 1 samples than cluster 2 samples.

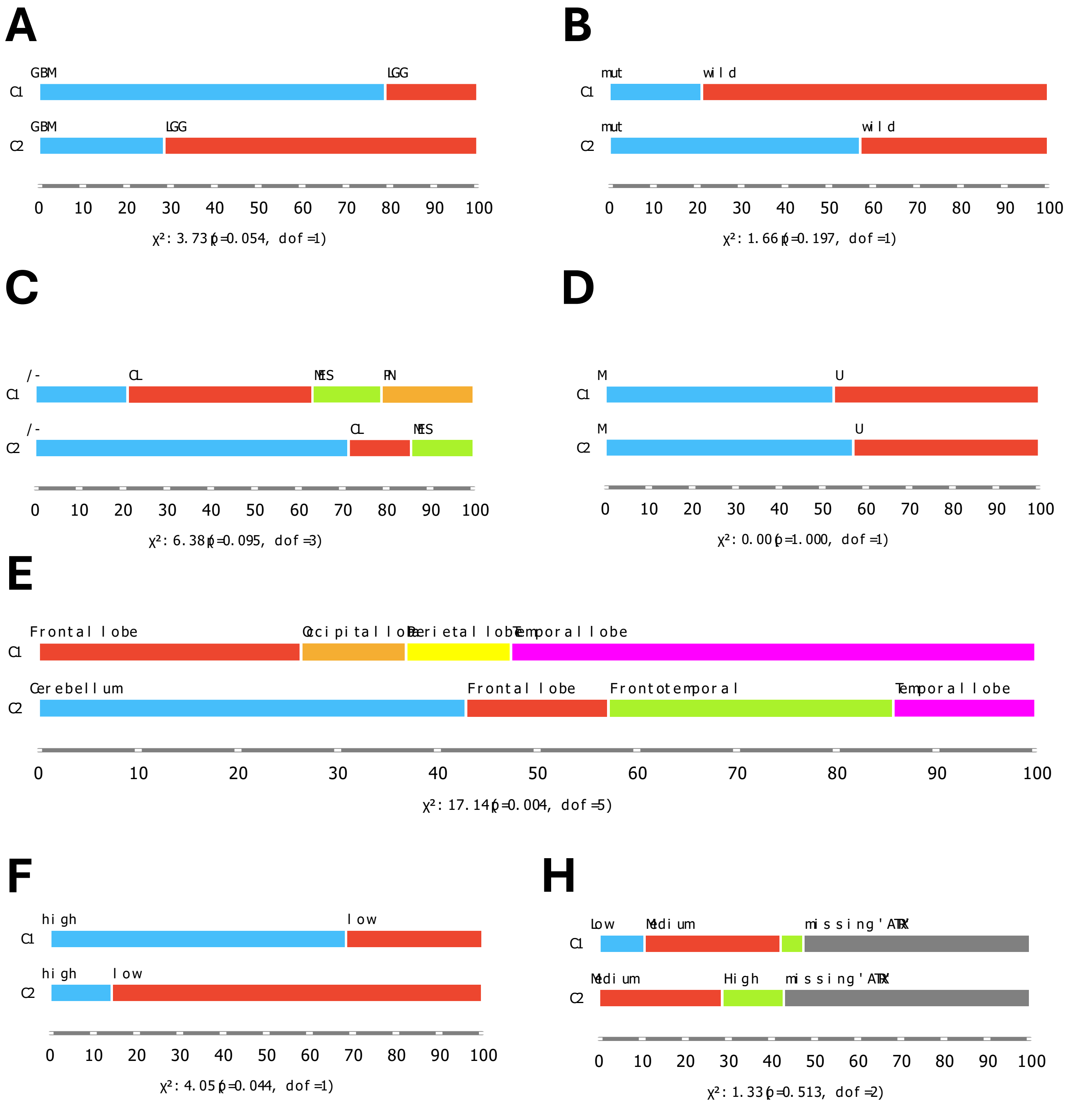

Fig. S5. Comparison of clinical and molecular features distribution between clusters (C1 vsC2). Statistics calculated applying chi-square analysis. (**A**) Tumor type distribution; (**B**) IDH1 mutation status distribution; (**C**) Glioblastoma molecular subtype distribution: CL –classical, MES – mesenchymal, PN – proneural, /- LGG samples, no subtype; D) MGMT methylation status distribution; (**E**) Tumor location distribution; (**F**) Ki67 index (indicates aggressiveness of gliomas) distribution; (**H**) ATRX level distribution.

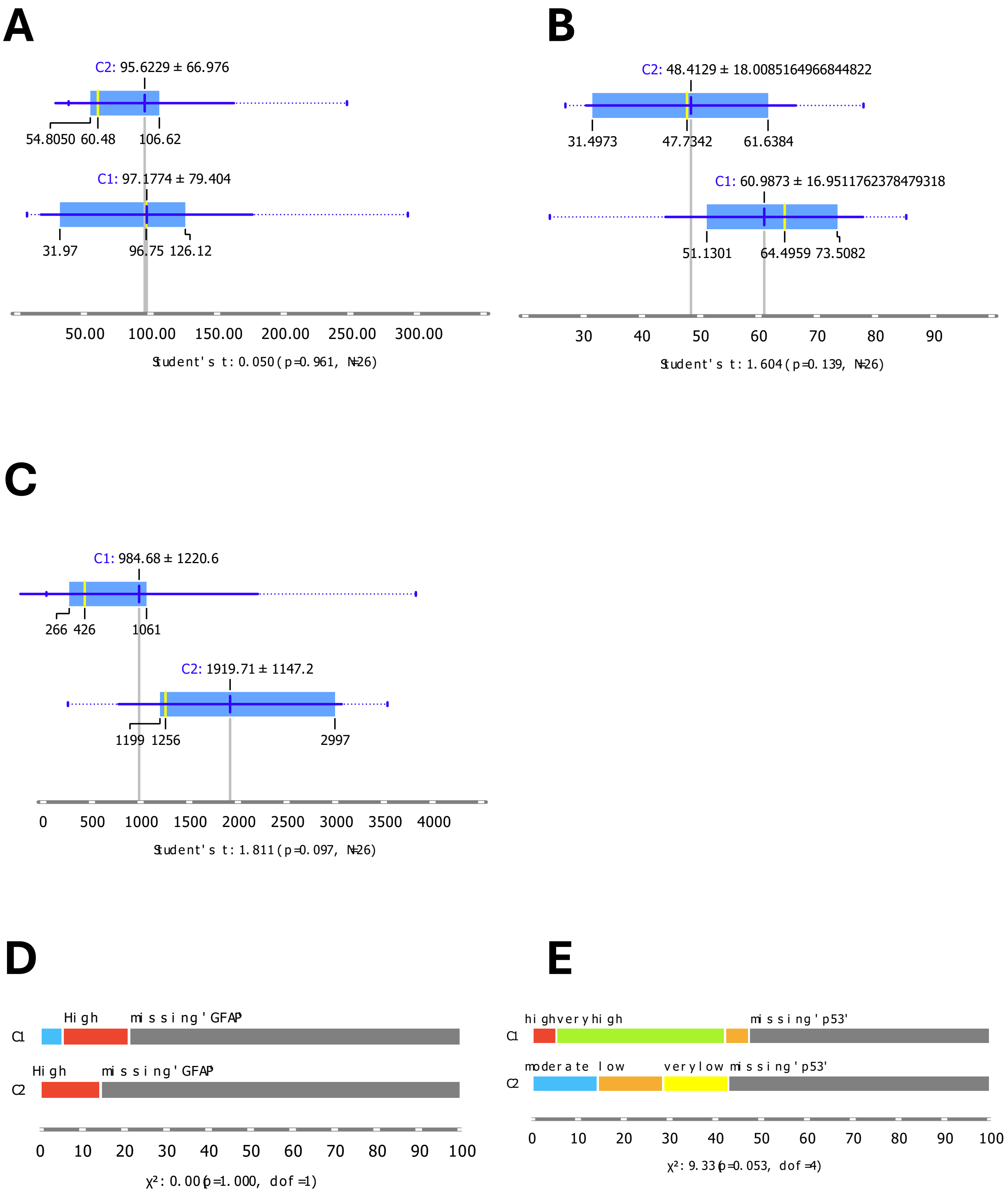

Fig. S6. Comparison of clinical and molecular features distribution between clusters (C1 vsC2). Statistics calculated applying Student's t-test. (**A**) Distribution of patient Age at the date of operation; (**B**) Tumor size distribution (cm3); (**C**) Patient survival distribution (days); (**D**) GFAP level distribution; (**E**) P53 level distribution.

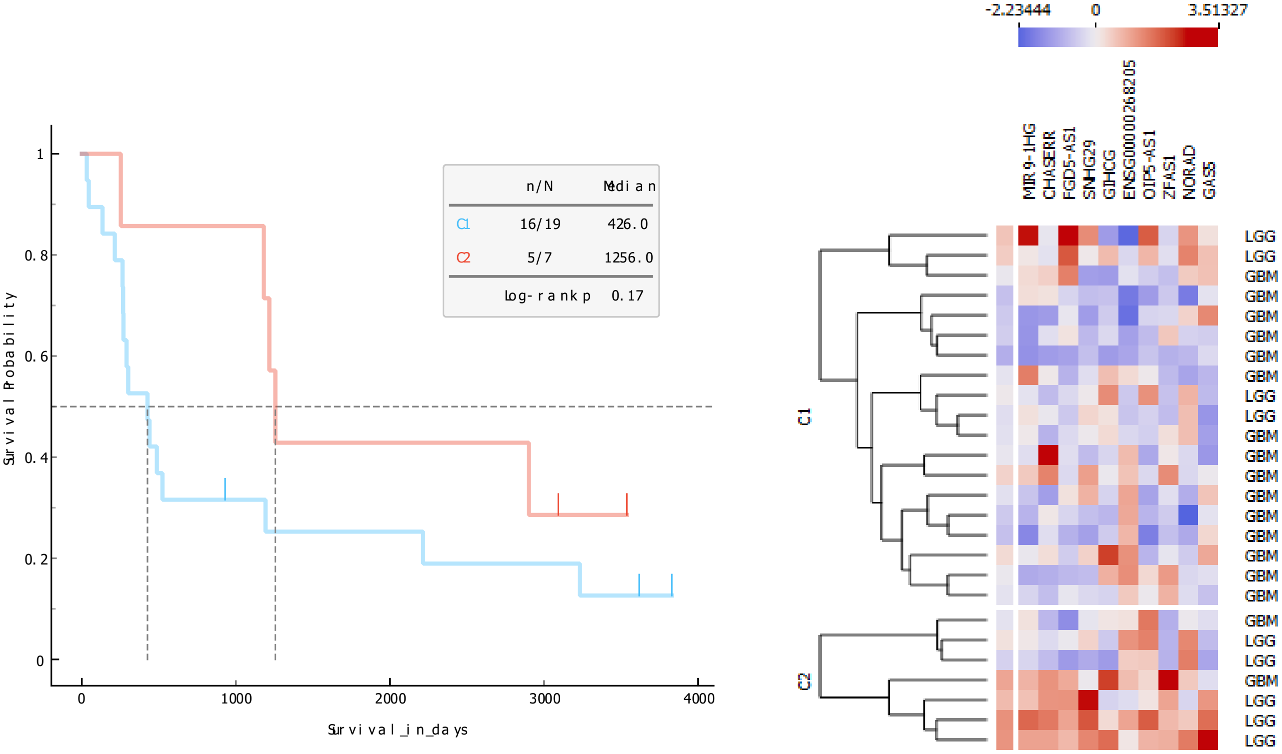

Fig. S7. (**A**) Kaplan-Meier survival curve of pateint from C1 and C2 clusters (log-rank p=0.17; df=1; X2=1.88). (**B**) Heat-map of selected 10 lncRNAs gene expression (selected based on m6A methylation). Values given as TPM standartized to μ=0, σ²=1.

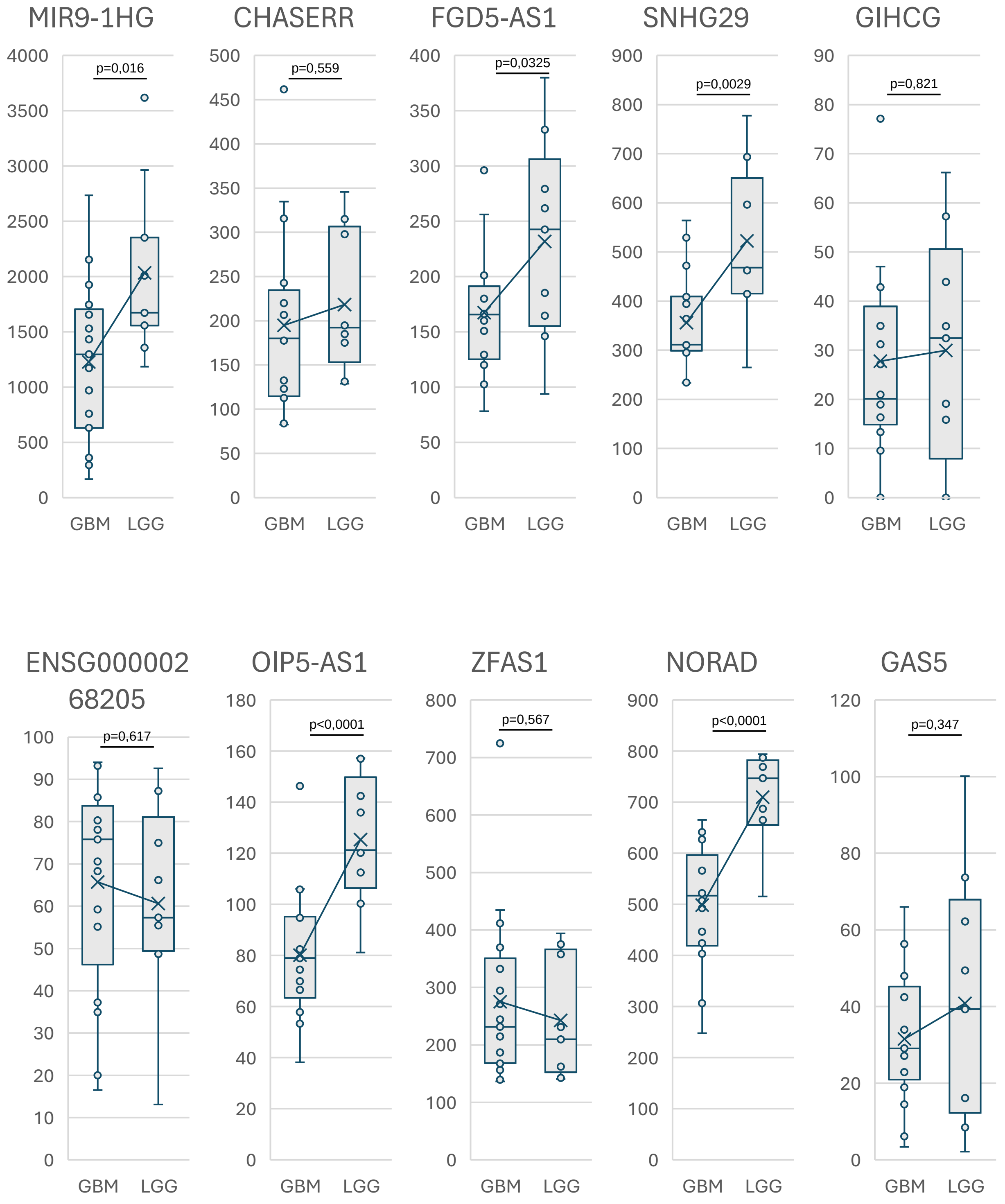

Fig. S8. Box-plot visualization of selected lncRNAs expression (TPM) level comparison between glioblastoma and low-grade glioma cohorts (t-test statistics).

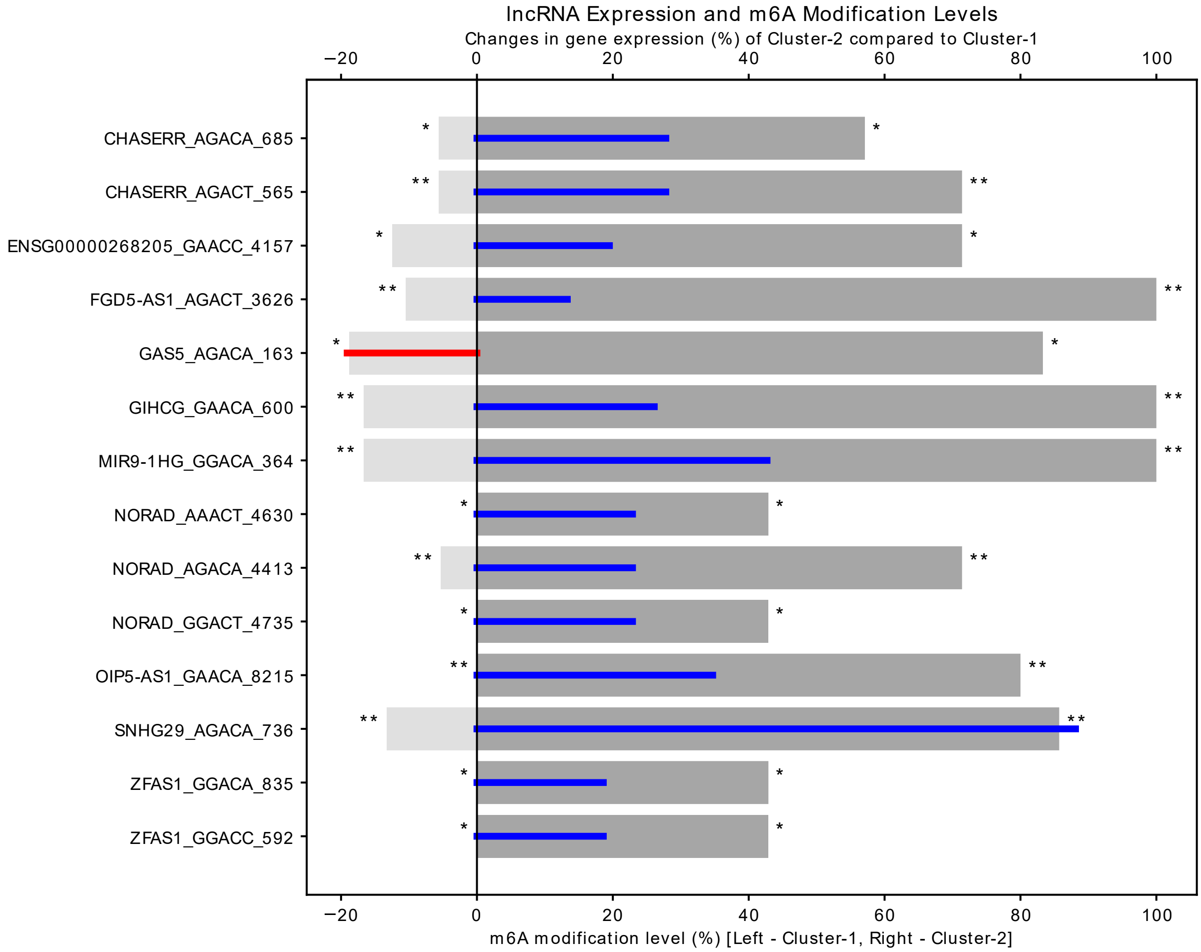

Fig. S9. RRACHs that were selected to be informative to distinguish between cluster m6A methylation (grey bars) and same lncRNAs expression (blue and red bars). Scale from 0 to 100 indicated increasing m6A and/or expression level in cluster 2 (C2), scale from 0 to -100 indicates increasing m6A and/or expression level in cluster 1 (C1). Asterisk indicated significant increase of methylation or expression in C1 or C2 accordingly.

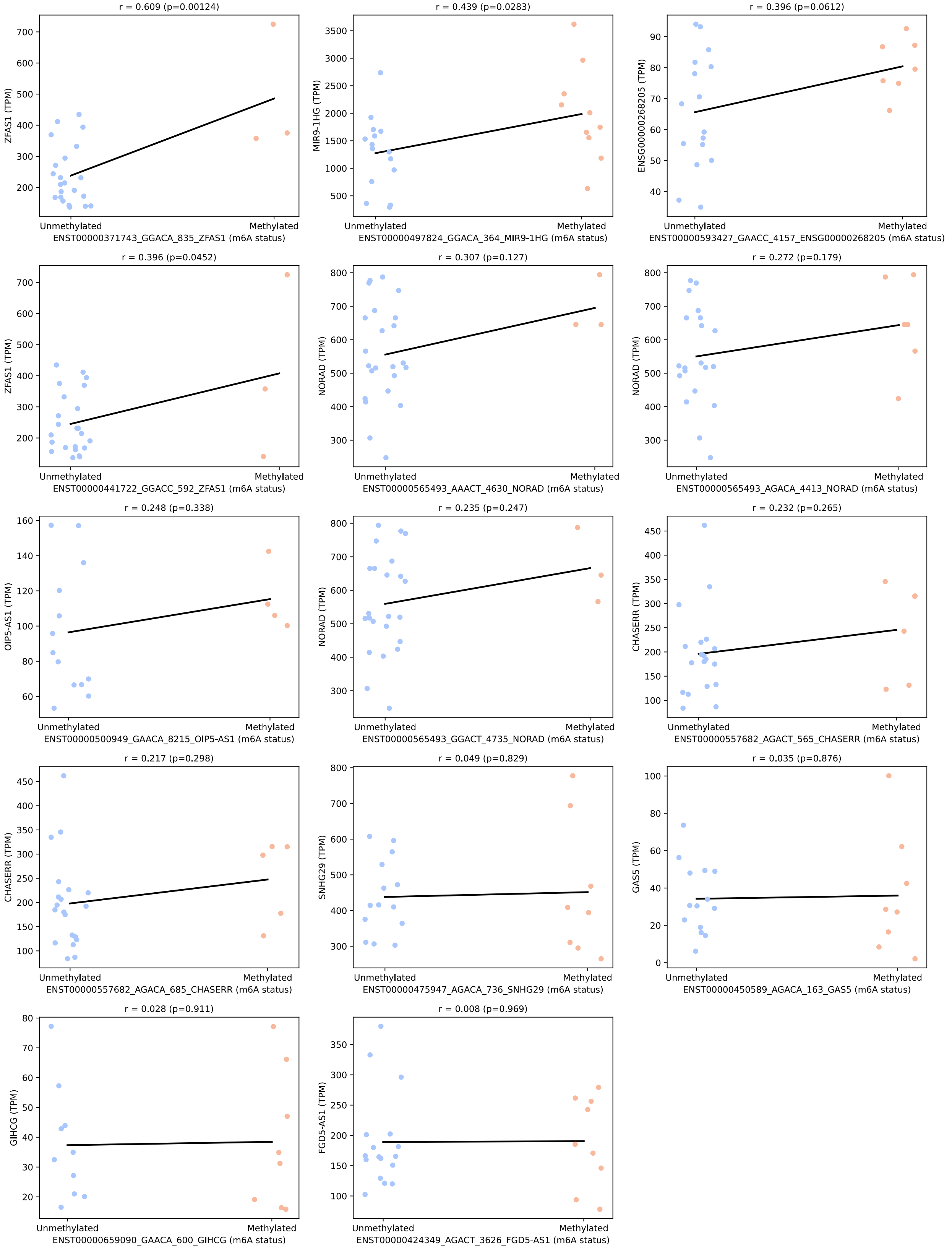

Fig. S10. Association between specific gene's m6A methylation site and the same gene expression. Strip plots depict the relationship between m6A methylation status (Unmethylated vs. Methylated) and gene expression (TPM values) at multiple m6A-gene combinations. Each subplot represents a different m6A location, with individual data points indicating gene expression levels across samples. Horizontal black lines connect group means to illustrate differences in expression between methylation states. The Point-Biserial Correlation coefficient (r) and p-value are displayed in each subplot, quantifying the strength and significance of the association.

Table St3. Pearsons correlation between m6A modulators and m6A modifications among different species of RNAs.

Correlation	FDR	Feature 1	Feature 2
-0.338	0.61292	ALKBH5	M6A Methylation%_pseudogenes
-0.292	0.62845	ALKBH5	M6A Methylation%_mRNAs
-0.221	0.69337	ALKBH5	M6A Methylation%_lncRNAs
-0.086	0.87324	ALKBH5	M6A Methylation%_other
0.226	0.69337	FTO	M6A Methylation%_lncRNAs
0.304	0.62845	FTO	M6A Methylation%_pseudogenes
0.313	0.62845	FTO	M6A Methylation%_mRNAs
0.368	0.5436	FTO	M6A Methylation%_other
0.074	0.87324	METTL14	M6A Methylation%_pseudogenes
0.083	0.87324	METTL14	M6A Methylation%_other
0.173	0.75096	METTL14	M6A Methylation%_mRNAs
0.18	0.7482	METTL14	M6A Methylation%_lncRNAs
-0.232	0.69337	METTL16	M6A Methylation%_other
0.038	0.92425	METTL16	M6A Methylation%_mRNAs
0.059	0.88726	METTL16	M6A Methylation%_pseudogenes
0.076	0.87324	METTL16	M6A Methylation%_lncRNAs
-0.253	0.69337	METTL3	M6A Methylation%_pseudogenes
-0.145	0.82967	METTL3	M6A Methylation%_mRNAs
-0.117	0.87324	METTL3	M6A Methylation%_lncRNAs
0.049	0.90214	METTL3	M6A Methylation%_other
0.044	0.91385	METTL5	M6A Methylation%_other
0.069	0.87324	METTL5	M6A Methylation%_lncRNAs
0.092	0.87324	METTL5	M6A Methylation%_mRNAs
0.27	0.66566	METTL5	M6A Methylation%_pseudogenes
0.09	0.87324	VIRMA	M6A Methylation%_other
0.177	0.7482	VIRMA	M6A Methylation%_mRNAs
0.248	0.69337	VIRMA	M6A Methylation%_lncRNAs
0.289	0.62845	VIRMA	M6A Methylation%_pseudogenes
-0.275	0.66152	WTAP	M6A Methylation%_pseudogenes
-0.223	0.69337	WTAP	M6A Methylation%_lncRNAs
-0.089	0.87324	WTAP	M6A Methylation%_mRNAs
-0.003	0.9924	WTAP	M6A Methylation%_other
-0.07	0.87324	ZCCHC4	M6A Methylation%_other
0.453	0.30643	ZCCHC4	M6A Methylation%_lncRNAs
0.464	0.30643	ZCCHC4	M6A Methylation%_mRNAs
0.58	0.08466	ZCCHC4	M6A Methylation%_pseudogenes

M6A Methylation% indicates the percentage of methylated RRACH motifs out of the total identified RRACH motifs

M6A Methylation%_other encompass miscRNA, mtRNA, scRNA, snRNA, IG_C_genes RNA, etc. This group represents very small part of all the identified RRACHs (from 0.002% to 0.14%).

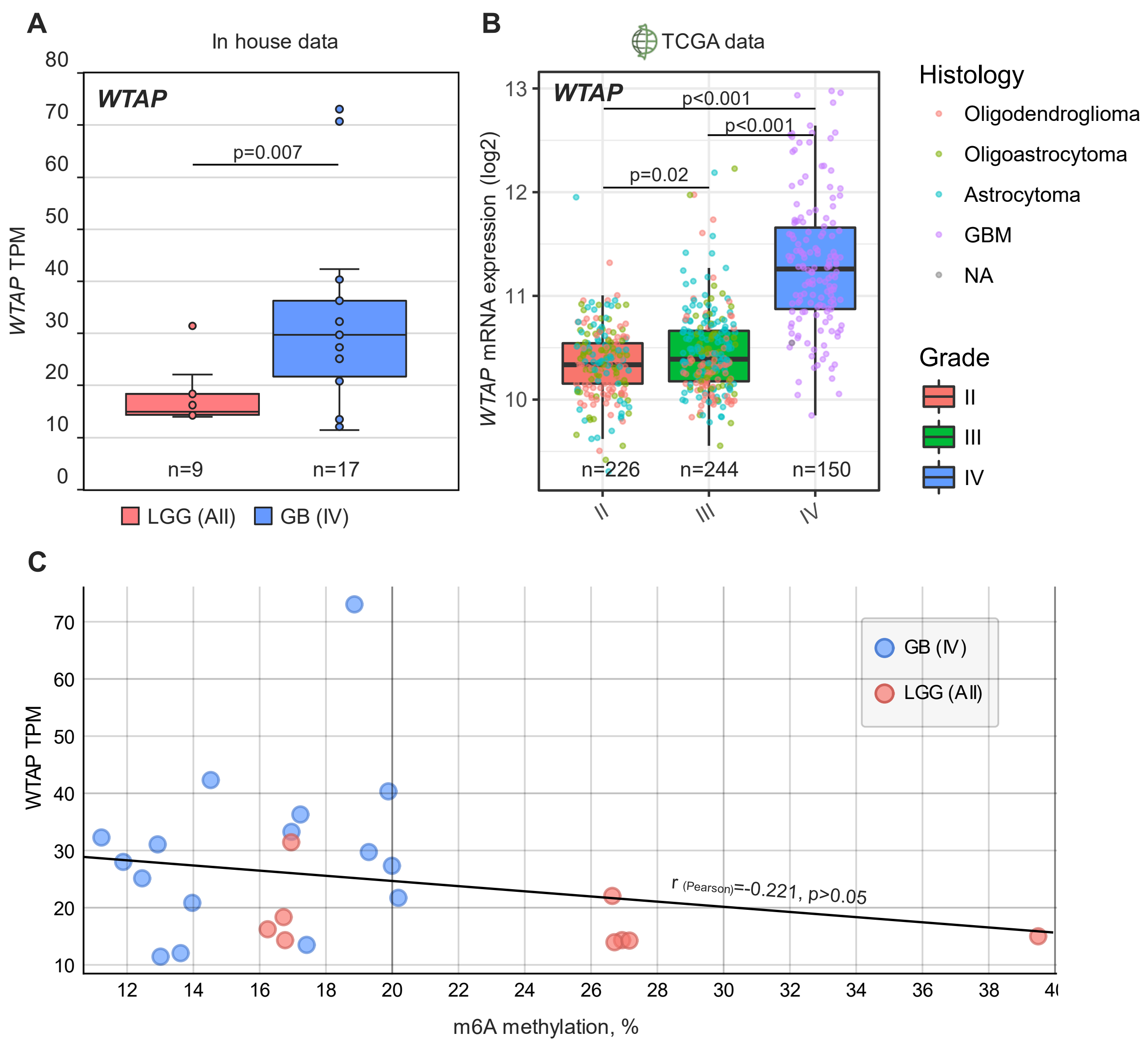

Fig. S11. Comparison of m6A writer WTAP mRNA levels between grade II glioma (red) and GBM (blue) in our in-house cohort (**A**) and in the TCGA cohort (**B**). Both datasets show increased WTAP expression in GBM. (**C**) Correlation between global (polyadenylated RNAs) RNAs m6A methylation and WTAP mRNA expression in human brain gliomas.

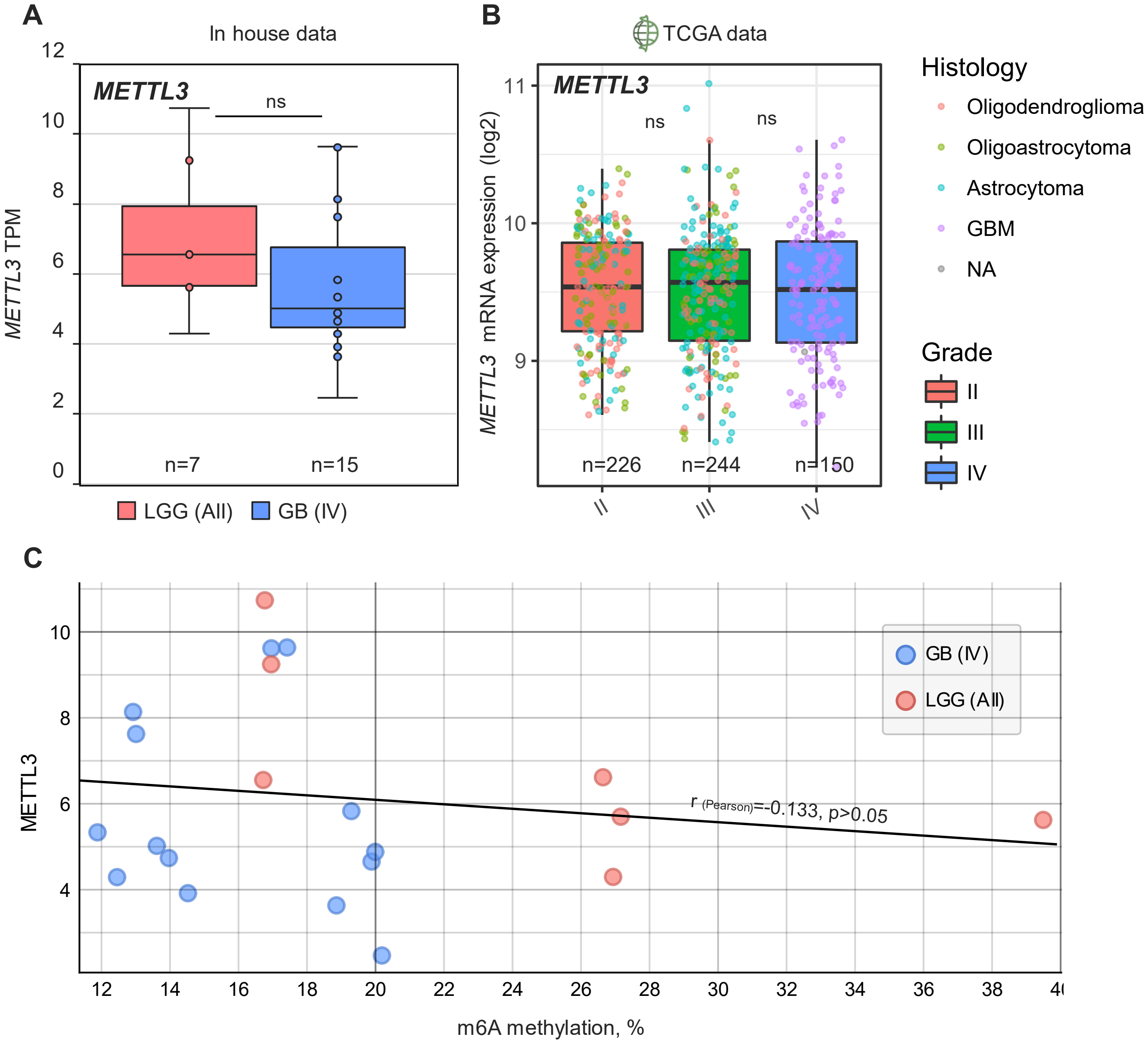

Fig. S12. Comparison of m6A writer METTL3 mRNA levels between grade II glioma (red) and GBM (blue) in our in-house cohort (**A**) and in the TCGA cohort (**B**). Both datasets show uniform METTL3 expressions between glioma malignancies. (**C**) Correlation between global (polyadenylated RNAs) RNAs m6A methylation and METTL3 mRNA expression in human brain gliomas.

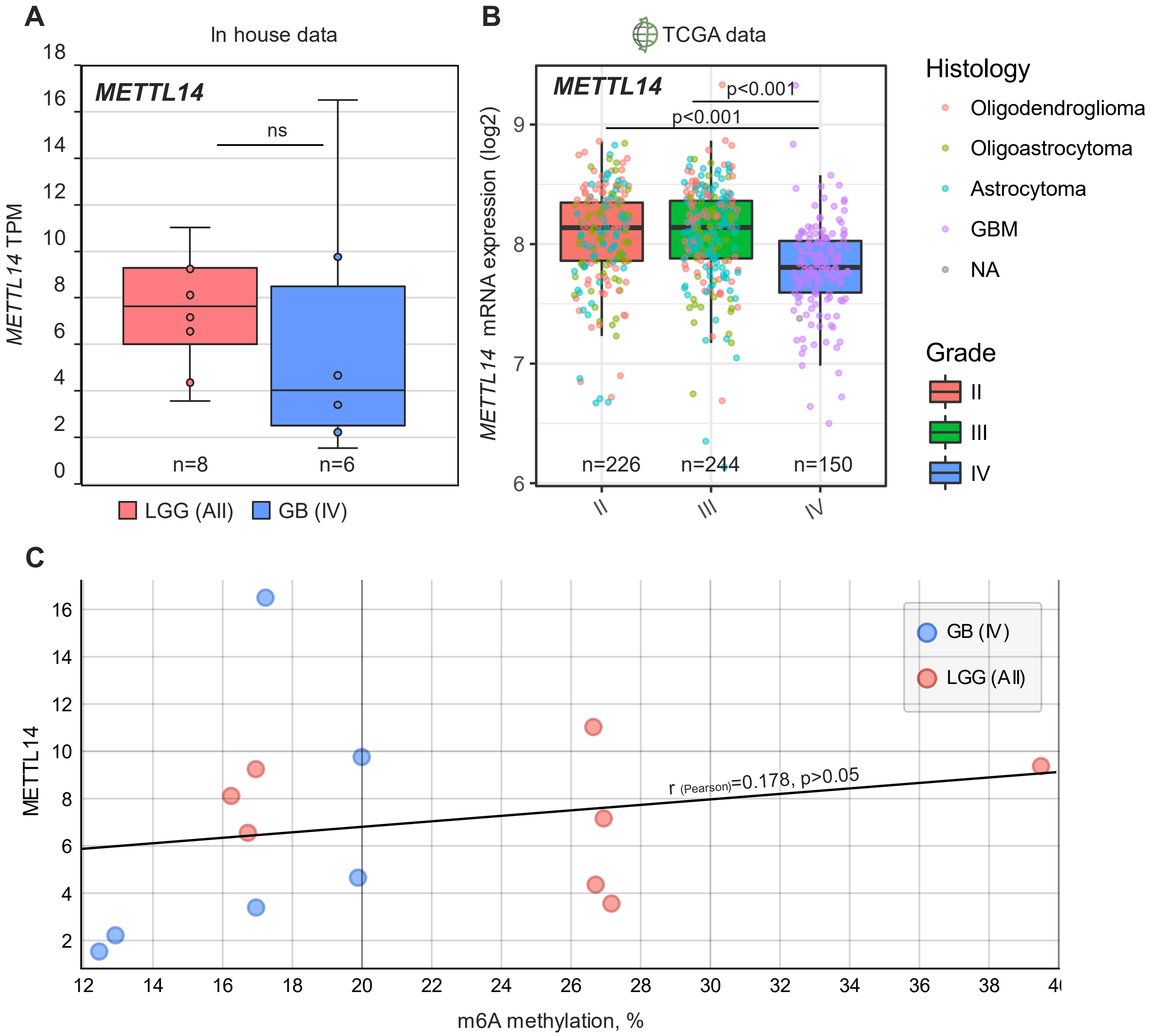

Fig. S13. Comparison of m6A writer METTL14 mRNA levels between grade II glioma (red) and GBM (blue) in our in-house cohort (**A**) and in the TCGA cohort (**B**). The TCGA dataset shows decreased METTL14 expression in GBM, while our in-house dataset lacks the data to confirm the same results. However, the trend remains consistent. (**C**) Correlation between global (polyadenylated RNAs) RNAs m6A methylation and METTL14 mRNA expression in human brain gliomas.

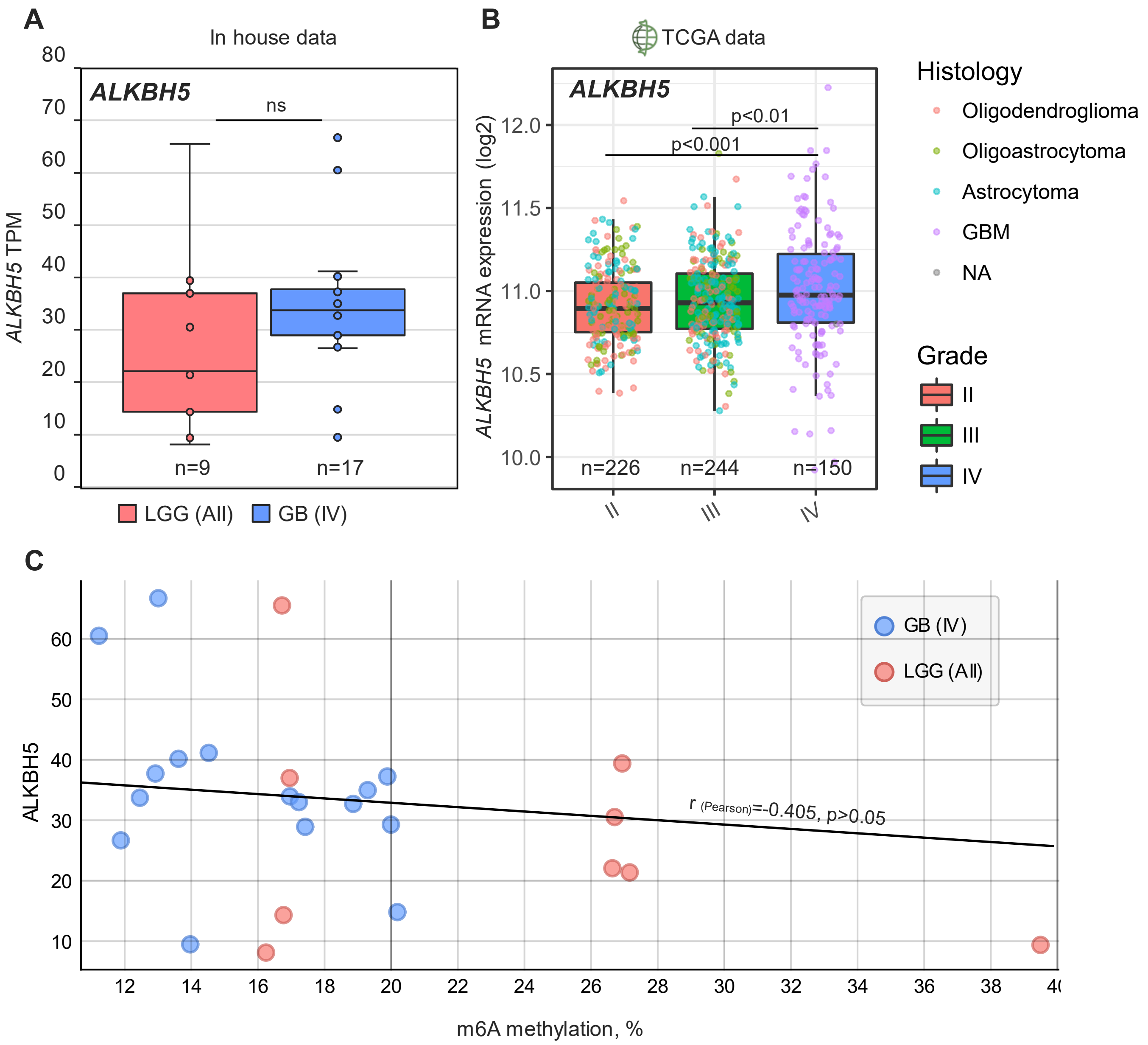

Fig. S14. Comparison of m6A eraser ALKBH5 mRNA levels between grade II glioma (red) and GBM (blue) in our in-house cohort (**A**) and in the TCGA cohort (**B**). The TCGA dataset shows slightly increased ALKBH5 expression in GBM, while our in-house dataset shows uniform expression between malignancies. However, the trend remains consistent. (**C**) Correlation between global (polyadenylated RNAs) RNAs m6A methylation and ALKBH5 mRNA expression in human brain gliomas.

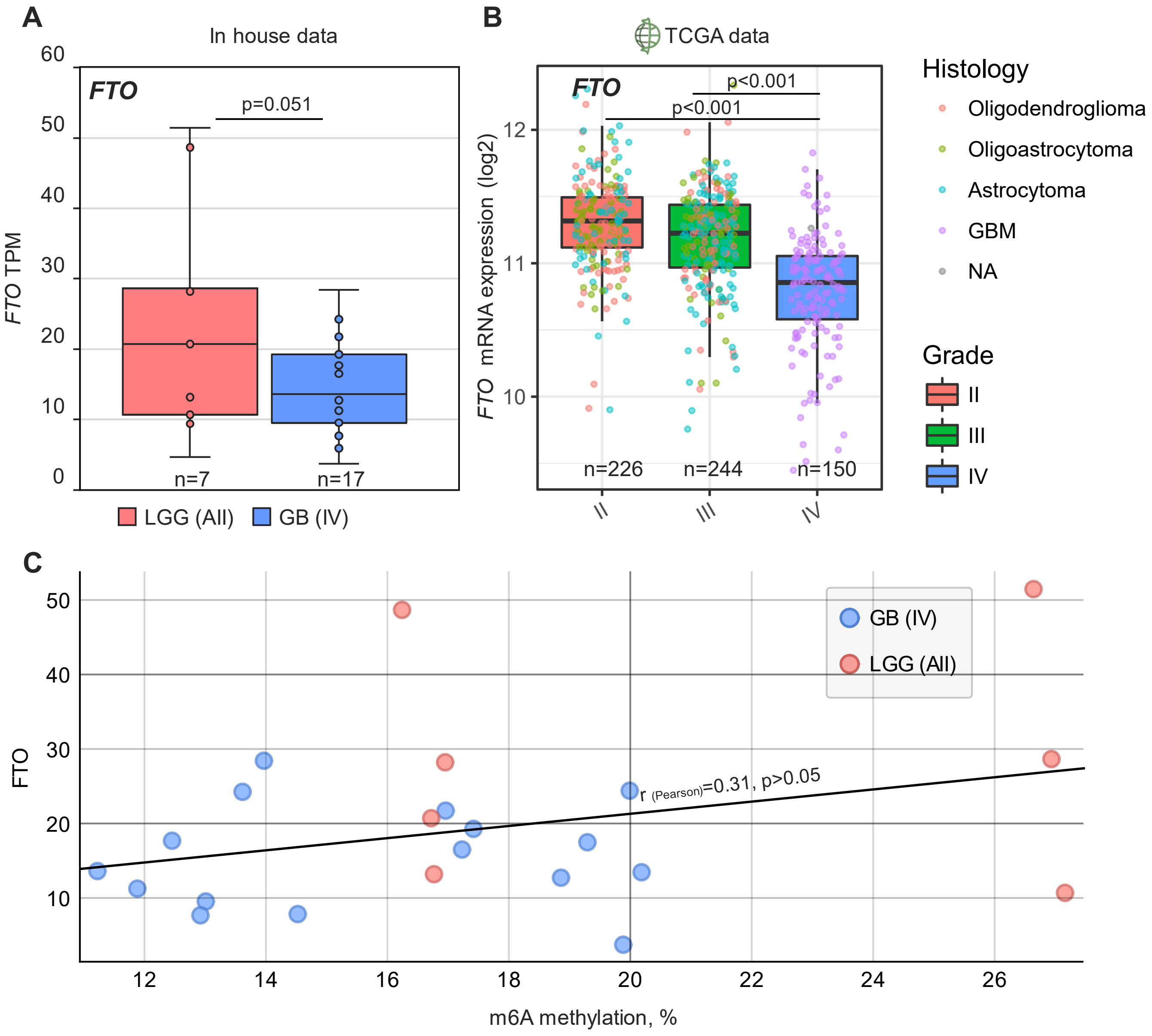

Fig. S15. Comparison of m6A eraser FTO mRNA levels between grade II glioma (red) and GBM (blue) in our in-house cohort (**A**) and in the TCGA cohort (**B**). Both dataset shows decreased FTO expression in GBMs as compared to grade II gliomas. (**C**) Correlation between global (polyadenylated RNAs) RNAs m6A methylation and FTO mRNA expression in human brain gliomas.

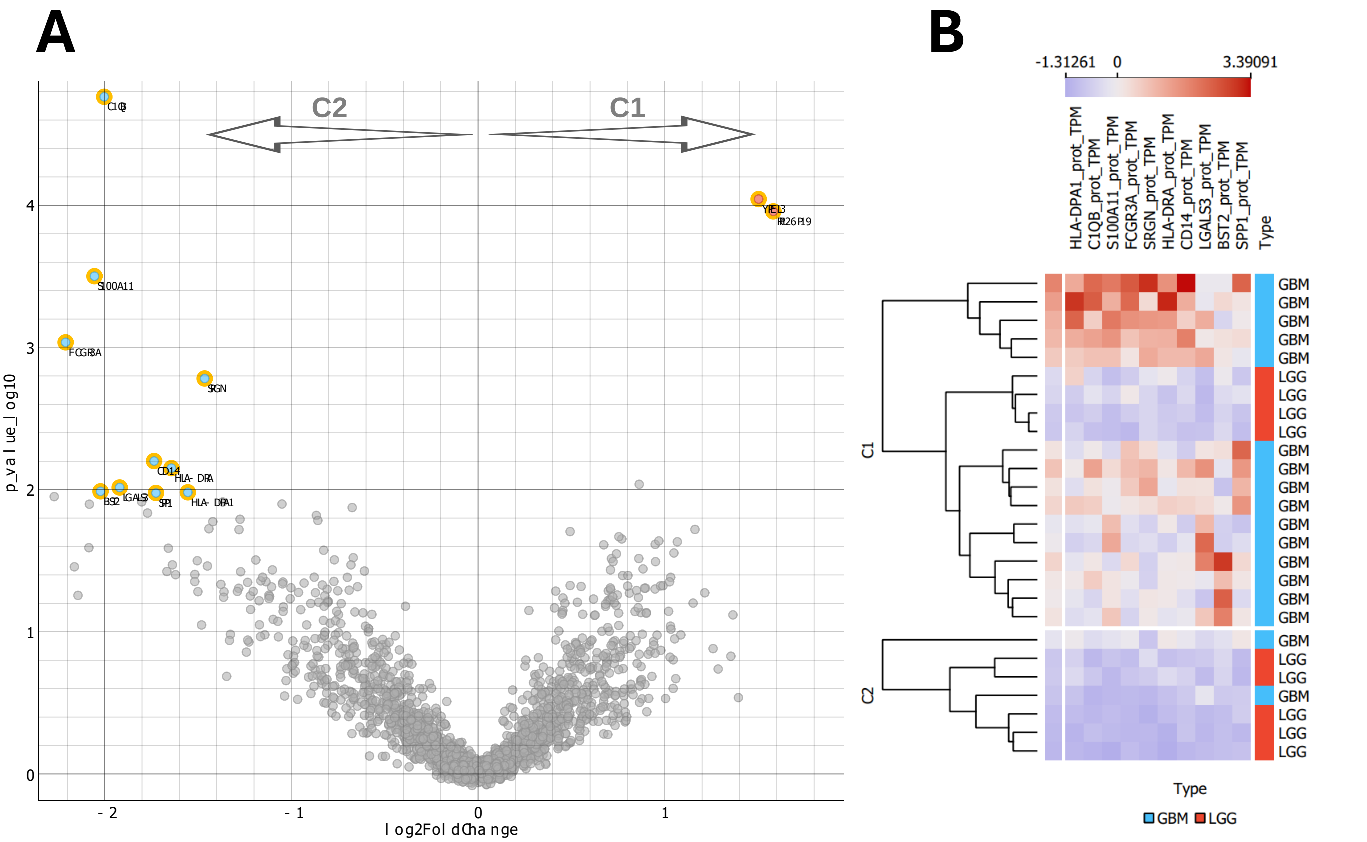

Fig. S16. (**A**) Deseq2 of mRNA between cluster C1 and C2. (**B**) Heat-map of 10 C1 cluster associated mRNA genes. Values given as TPM standartized to μ=0, σ²=1.

Table St4. GO analysis of 10 genes upregulated in C1 cluster samples. Output of: biological procese.

Organism: Human		Annotation provider: GOA			
**GOID**	**Term**	**P-value**	**Uncorrected P-value**	**Number annotated**	**Annotated genes**
GO:0002682	regulation of immune system process	4.93117e-06	2.03767e-08	7	BST2, C1QB, CD14, FCGR3A, HLA-DPA1, HLA-DRA, LGALS3
GO:0051239	regulation of multicellular organismal process	8.63673e-06	3.5689e-08	8	BST2, CD14, FCGR3A, HLA-DPA1, HLA-DRA, LGALS3, SPP1, SRGN
GO:0045087	innate immune response	1.02385e-05	4.23078e-08	6	BST2, C1QB, CD14, FCGR3A, HLA-DPA1, LGALS3
GO:0050776	regulation of immune response	1.39533e-05	5.76581e-08	6	C1QB, CD14, FCGR3A, HLA-DPA1, HLA-DRA, LGALS3
GO:0140546	defense response to symbiont	1.82706e-05	7.54984e-08	6	BST2, C1QB, CD14, FCGR3A, HLA-DPA1, LGALS3
GO:0002684	positive regulation of immune system process	2.61718e-05	1.08148e-07	6	C1QB, CD14, FCGR3A, HLA-DPA1, HLA-DRA, LGALS3
GO:0006955	immune response	3.15595e-05	1.30411e-07	7	BST2, C1QB, CD14, FCGR3A, HLA-DPA1, HLA-DRA, LGALS3
GO:0098542	defense response to other organism	4.28081e-05	1.76893e-07	6	BST2, C1QB, CD14, FCGR3A, HLA-DPA1, LGALS3
GO:0009605	response to external stimulus	6.6656e-05	2.75438e-07	7	BST2, C1QB, CD14, FCGR3A, HLA-DPA1, LGALS3, SPP1
GO:0070663	regulation of leukocyte proliferation	8.20806e-05	3.39176e-07	4	BST2, FCGR3A, HLA-DPA1, LGALS3
GO:0050896	response to stimulus	8.8679e-05	3.66442e-07	10	BST2, C1QB, CD14, FCGR3A, HLA-DPA1, HLA-DRA, LGALS3, S100A11, SPP1, SRGN

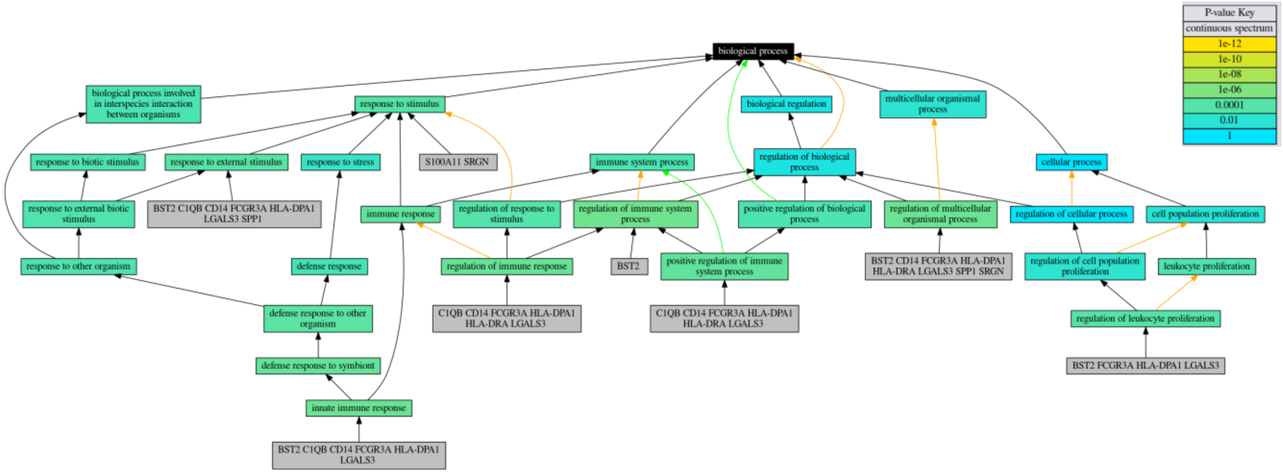

Fig. S17. GO analysis for biological proceses output map (10 genes upregulated in C1 cluster samples).

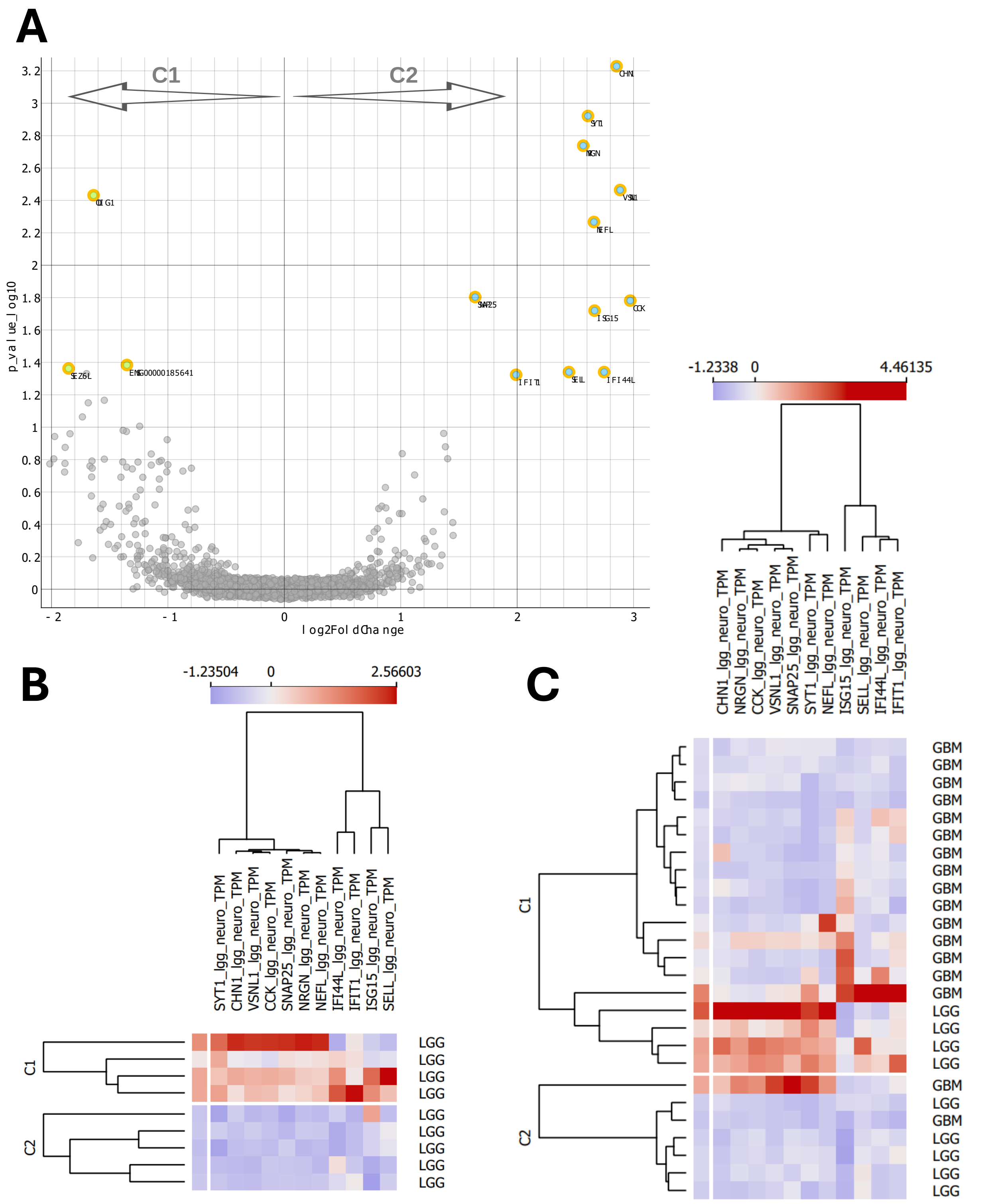

Fig. S18. (**A**) Deseq2 of mRNA between LGGs at cluster C1 and C2. (**B**) Heat-map of 11 C1 cluster associated mRNA genes in LGGs only, (**C**) in whole cohort analyzed. Values given as TPM standartized to μ=0, σ²=1.

Table St5. GO analysis of 11 genes upregulated in LGGs C1 cluster. Output of: biological proceses

Organism: Human		Annotation provider: GOA			
GOID	Term	P-value	Uncorrected P-value	Number annotated	Annotated genes
GO:0048812	neuron projection morphogenesis	6.39615e-05	3.15081e-07	5	CCK, CHN1, NEFL, SNAP25, SYT1
GO:0120039	plasma membrane bounded cell projection morphogenesis	7.18925e-05	3.5415e-07	5	CCK, CHN1, NEFL, SNAP25, SYT1
GO:0048858	cell projection morphogenesis	7.45247e-05	3.67117e-07	5	CCK, CHN1, NEFL, SNAP25, SYT1
GO:0000902	cell morphogenesis	0.000503039	2.47802e-06	5	CCK, CHN1, NEFL, SNAP25, SYT1
GO:0031175	neuron projection development	0.000580006	2.85717e-06	5	CCK, CHN1, NEFL, SNAP25, SYT1
GO:0050896	response to stimulus	0.00064933	3.19867e-06	10	CCK, CHN1, IFI44L, IFIT1, ISG15, NEFL, NRGN, SELL, SYT1, VSNL1
GO:0007409	axonogenesis	0.000694631	3.42183e-06	4	CCK, CHN1, NEFL, SNAP25
GO:0048791	calcium ion-regulated exocytosis of neurotransmitter	0.000736786	3.62949e-06	2	SNAP25, SYT1

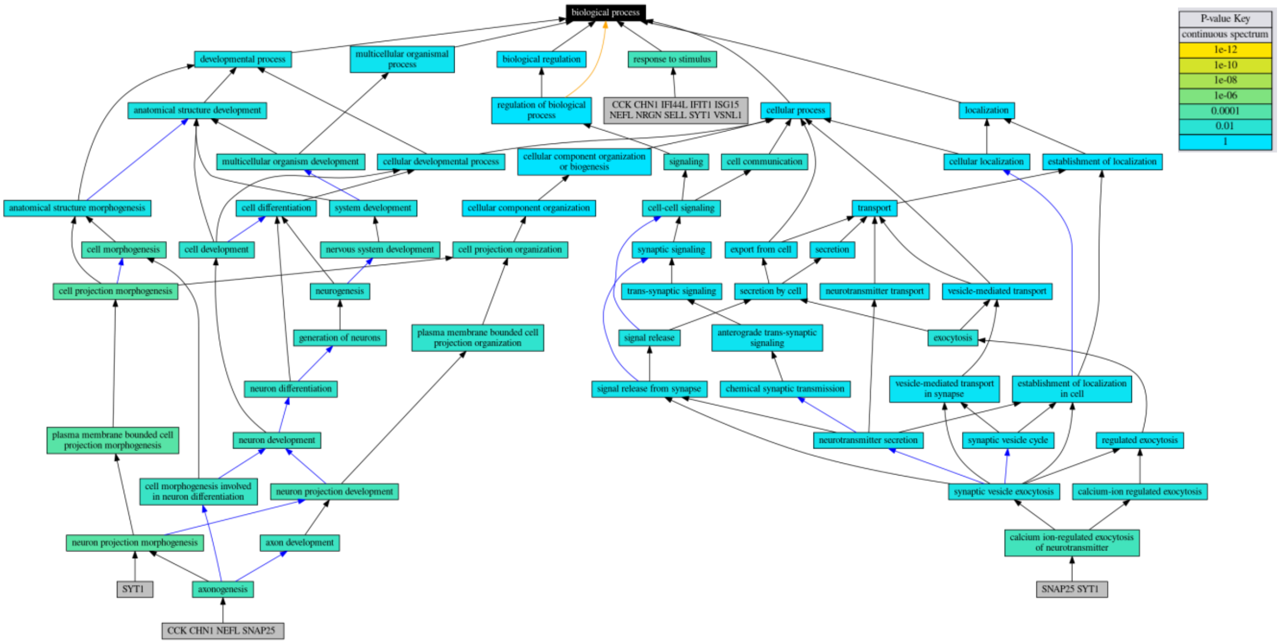

Fig. S19. GO analysis for biological proceses output map (11 genes upregulated in LGGs C1 cluster).

Fig. S20. **(A)** Barplot illustrating the length and error rate of the RNA sequences. The first subplot shows a median read length that was sequenced. In general, no outliers are seen, which indicates that, during RNA extraction and direct RNA sequencing library preparation steps, all samples had a similar RNA fragmentation rate. Although higher variation is seen in a glioblastoma group. The second subplot shows the basecalling accuracy. Up to date, it is usual for direct RNA sequencing to produce lower basecalling accuracy, compared to DNA or non-direct RNA sequencing. A small variation between the height of each bar, and an expected basecalling score around 10, indicates that there were no major contaminants in the samples. A quality score of 10 means that there is a 1 out of 10 chance that a sequenced nucleotide was identified incorrectly. The third subplot shows the standard deviation of a read length for each sample.
(B) Lineplot illustrating gene body coverage. The figure shows which parts of the gene were sequenced more often. This type of figure helps to better estimate RNA fragmentation. The x-axis represents a sequenced percentage of a total gene's length. Meaning that 0% of a gene body indicates the very start of a gene, or its 5'-end, while 100% of a gene body is the very end of a gene, or its 3'-end. Since direct RNA sequencing is performed from 3'-end to 5'-end, it is expected to have a higher coverage at the 3'-end of a gene. Higher verticality of a line indicates higher RNA fragmentation. A more horizontal line indicates that a higher amount of intact, full-length RNAs were sequenced. Each line in this figure indicates a unique sample - 17 glioblastoma, and 9 low-grade glioma samples.

A

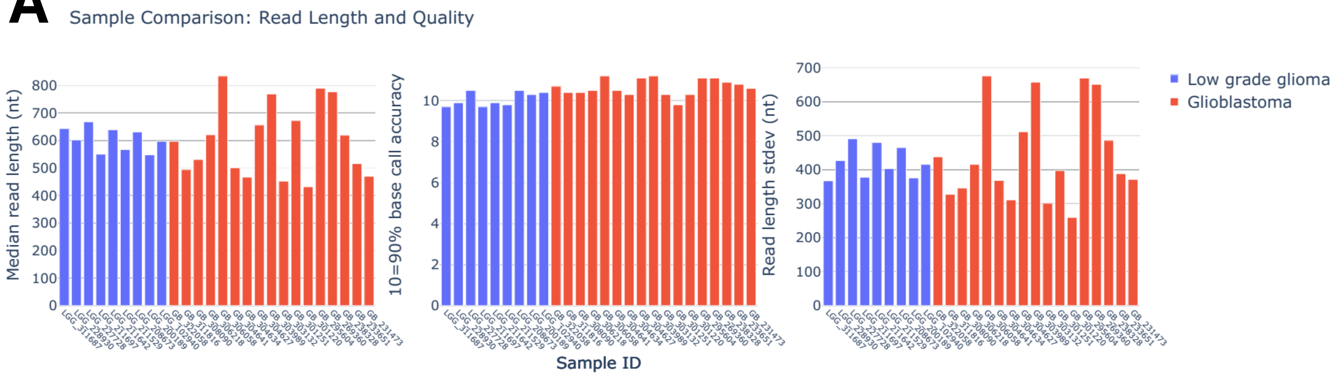

B

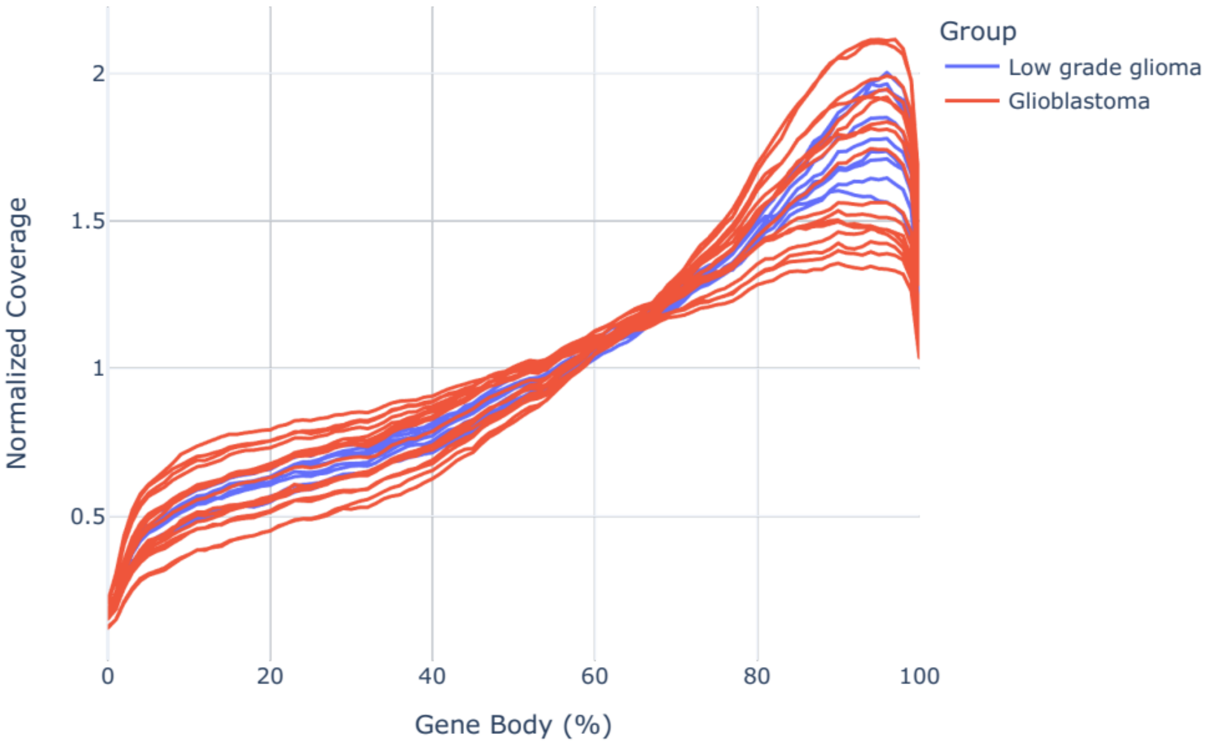

Supplement: Supplementary file 1 — Supplementary Material 1. [file 10020_2025_1254_MOESM1_ESM.pdf]
